# Supplementary material for: Accelerated discovery of multi-elemental reverse water-gas shift catalysts using extrapolative machine learning approach
Source: Nat Commun. 2023 Sep 21;14:5861. doi: 10.1038/s41467-023-41341-3 (PMC10514199; doi:10.1038/s41467-023-41341-3)
Supplement: Supplementary file 1 — Supplementary Information [file 41467_2023_41341_MOESM1_ESM.docx]

Supplementary Information

**Accelerated discovery of multi-elemental reverse water-gas shift catalysts using extrapolative machine learning approach**

Gang Wang,^1†^ Shinya Mine,^1†^ Duotian Chen,^1†^ Yuan Jing,^1^ Kah Wei Ting,^1^ Taichi Yamaguchi,^1^ Motoshi Takao,^1^ Zen Maeno,^2^ Ichigaku Takigawa,*^3,4,5^ Koichi Matsushita,^6^ Ken-ichi Shimizu,*^1^ Takashi Toyao*^1^

^1^ Institute for Catalysis, Hokkaido University, N-21, W-10, Sapporo 001-0021, Japan

^2^ School of Advanced Engineering, Kogakuin University, 2665-1, Nakano-cho, Hachioji 192-0015, Japan

^3^ RIKEN Center for Advanced Intelligence Project, 1-4-1 Nihonbashi, Chuo-ku, Tokyo 103-0027, Japan

^4^ Institute for Chemical Reaction Design and Discovery (WPI-ICReDD), Hokkaido University, N-21, W-10, Sapporo 001-0021, Japan

^5^ Institute for Liberal Arts and Sciences, Kyoto University, 69-302, Yoshida-Konoe-cho, Sakyo-ku, Kyoto 606-8315, Japan

^6^ Central Technical Research Laboratory, ENEOS Corporation, 8, Chidori-cho, Naka-ku, Yokohama 231-0815, Japan

†These authors contributed equally to this work.

*Corresponding author Email: takigawa.ichigaku.8s@kyoto-u.ac.jp (I.T.), kshimizu@cat.hokudai.ac.jp (K.S.), toyao@cat.hokudai.ac.jp (T.T.)

**Supplementary Methods**

**Supplementary Table 1.** Sources and purity of chemicals used to prepare catalysts.

| AN | Symbol | Chemical formula | Purity/concentration | Company name |
| --- | --- | --- | --- | --- |
| 3 | Li | LiNO_3_ | 99.99% | Kanto Chemical |
| 11 | Na | NaNO_3_ | 99.99% | Aldrich |
| 12 | Mg | Mg(NO_3_)_2_ | 99.99% | Kanto Chemical |
| 13 | Al | Al(NO_3_)_3_・9H_2_O | 99.99% | Wako Chemical |
| 14 | Si | Si(OCH_2_CH_3_)_4_ | 97% | Tokyo Chemical Industry |
| 19 | K | KNO_3_ | 99.99% | Wako Chemical |
| 20 | Ca | Ca(NO_3_)_2_・4H_2_O | 98.5% | Wako Chemical |
| 21 | Sc | Sc(OOCCH_3_)_3_・nH_2_O | 98% | Tokyo Chemical Industry |
| 22 | Ti | C_4_H_10_N_2_O_10_Ti | 99.99% | Alfa Aesar |
| 23 | V | NH_4_VO_3_ | 99.99% | Wako Chemical |
| 24 | Cr | Cr(NO_3_)_3_・9H_2_O | 98.5% | Aldrich |
| 25 | Mn | Mn(NO_3_)_2_・6H_2_O | 99.99% | Wako Chemical |
| 26 | Fe | Fe(NO_3_)_3_・9H_2_O | 98% | Aldrich |
| 27 | Co | Co(NO_3_)_2_・6H_2_O | 99.99% | Wako Chemical |
| 28 | Ni | Ni(NO_3_)_2_・6H_2_O | 98% | Wako Chemical |
| 29 | Cu | Cu(NO_3_)_2_・3H_2_O | 99.99% | Aldrich |
| 30 | Zn | Zn(NO_3_)_2_・6H_2_O | 99.99% | Kanto Chemical |
| 31 | Ga | Ga(NO_3_)_3_・8H_2_O | 99.99% | Wako Chemical |
| 32 | Ge | (NH_4_)_2_GeF_6_ | 99.99% | Wako Chemical |
| 37 | Rb | RbNO_3_ | 99.99% | Wako Chemical |
| 38 | Sr | Sr(NO_3_)_2_ | 98% | Kanto Chemical |
| 39 | Y | Y(NO_3_)_3_・6H_2_O | 99.99% | Mitsuwa Chemical |
| 40 | Zr | Zr(NO_3_)_2_O・2H_2_O | 99.99% | Mitsuwa Chemical |
| 41 | Nb | Nb(HC_2_O_4_)_5_・5H_2_O | 95% | Mitsuwa Chemical |
| 42 | Mo | (NH_4_)_6_Mo_7_O_24_・4H_2_O | 99.99% | Wako Chemical |
| 44 | Ru | Ru(NO)(NO_3_)_3_ | 1.5 wt% Ru aqueous solution | Strem Chemical |
| 45 | Rh | Rh(NO_3_)_3_ | 50 g/L Rh aqueous solution | Tanaka Chemical |
| 46 | Pd | Pd(NH_3_)_2_(NO_2_)_2_ | 50.5 g/L Pd aqueous solution | Kojima Chemical |
| 47 | Ag | AgNO_3_ | 99.99% | Wako Chemical |
| 48 | Cd | Cd(NO_3_)_2_・4H_2_O | 99% | Aldrich |
| 49 | In | In(NO_3_)_3_・3H_2_O | 99.99% | Kanto Chemical |
| 50 | Sn | SnCl_2_ | 99.99% | Kishida Chemical |
| 51 | Sb | Sb(OOCCH_3_)_3_ | 99.99% | Aldrich |
| 55 | Cs | CsNO_3_ | 99.99% | Wako Chemical |
| 56 | Ba | Ba(NO_3_)_2_ | 99.99% | Kanto Chemical |
| 57 | La | La(NO_3_)_3_・6H_2_O | 99.99% | Mitsuwa Chemical |
| 58 | Ce | Ce(NO_3_)_3_・6H_2_O | 99.99% | Wako Chemical |
| 59 | Pr | Pr(NO_3_)_3_・6H_2_O | 99.99% | Aldrich |
| 60 | Nd | Nd(NO_3_)_3_・6H_2_O | 99.5% | Wako Chemical |
| 62 | Sm | Sm(NO_3_)_3_・6H_2_O | 99.5% | Mitsuwa Chemical |
| 63 | Eu | Eu(NO_3_)_3_・6H_2_O | 99.99% | Alfa Aesar |
| 64 | Gd | Gd(NO_3_)_3_・6H_2_O | 99.5% | Wako Chemical |
| 65 | Tb | Tb(NO_3_)_3_・6H_2_O | 99.9% | Strem Chemicals |
| 66 | Dy | Dy(NO_3_)_3_・6H_2_O | 99.5% | Wako Chemical |
| 67 | Ho | Ho(NO_3_)_3_・nH_2_O | 99.5% | Wako Chemical |
| 68 | Er | Er(NO_3_)_3_・nH_2_O | 99.99% | Wako Chemical |
| 69 | Tm | Tm(NO_3_)_3_・5H_2_O | 99.99% | Mitsuwa Chemical |
| 70 | Yb | Yb(NO_3_)_3_・nH_2_O | 99.99% | Wako Chemical |
| 71 | Lu | Lu(NO_3_)_3_・nH_2_O | 99.99% | Wako Chemical |
| 72 | Hf | HfCl_4_ | 99.5% | Wako Chemical |
| 74 | W | (NH_4_)_10_W_12_O_41_・5H_2_O | 99.99% | Wako Chemical |
| 75 | Re | NH_4_ReO_4_ | 99.99% | Aldrich |
| 77 | Ir | IrCl_3_・3H_2_O | 99.99% | Kanto Chemical |
| 78 | Pt | Pt(NH_3_)(NO_2_)_2_ | 50 g/L Pt aqueous solution | Tanaka Chemical |
| 83 | Bi | Bi(NO_3_)_3_・5H_2_O | 99.99% | Wako Chemical |

**Catalyst Characterization**

X-ray diffraction (XRD) patterns were recorded using a Miniflex (Rigaku) with CuKα radiation. AUTOSORB 6AG (Yuasa Ionics Co.) was used for the N_2_ adsorption measurements. Temperature-programmed reduction with H_2_ (H_2_-TPR) was performed using a BELCAT II (MicrotracBEL) with a cryo apparatus. Samples (100 mg) were mounted in a cell and then heated at a ramping rate of 10 °C min^-1^ under a flow of 5% H_2_/Ar (20 mL min^-1^). The effluent gas passed through a trap system that contained MS4A to remove the produced water and then through a thermal conductivity detector (TCD) to determine the amount of H_2_ consumed in the process. Owing to the experimental setup, separate experiments were carried out over temperature ranges of -100–50 °C and 50–850 °C.

**ML Methods**

*E*_ads_ of CO_2_ as an elemental descriptor was calculated using the Vienna ab initio simulation package (VASP, version 5.4.4)^12^. The Perdew-Burke-Ernzerhof functional revised for solids (PBEsol) ^3^ was employed in combination with the projector-augmented wave (PAW) method.^4^ A kinetic energy cutoff of 400 eV was set for the plane-wave basis sets. Gaussian smearing with a width of 0.2 eV was applied for the occupation of the electronic levels. The Brillouin zone was sampled using 2×2×1 Monkhorst-Pack grids. Van der Waals interactions were described using the dispersion-corrected DFT-D3 (BJ) function.^5^ The convergence of force on each atom was set to 0.03 eV Å^-1^. We used the most stable and common metallic bulk structure and facet for each element. Detailed information is given on our GitHub (https://github.com/shinya-mine).

The eight selected descriptors (Supplementary Fig. 2 and Supplementary Table 2) showed the highest prediction accuracy. Other elemental descriptor candidates including atomic weight (AW), atomic radius (AR), EN of other definitions, boiling point (b.p.), ionization energy (IE), electron affinity (EA), and surface energy (SE) were also tested as an initial screening, but the combination of the eight descriptors specified above was found to give the best results.

For performance prediction, in addition to ETR, five well-established ML models were used: least absolute shrinkage and selection operator regression (Lasso)^6^, ridge regression (Ridge)^7^, Gaussian process regression (GPR)^8^, support vector regression (SVR)^9^, and random forest regression (RFR)^10^. Two of these methods involve linear regression methods (Lasso, Ridge), and the other four methods including ETR involve nonlinear regression methods. More specifically, two kernel methods (GPR, SVR) and two types of decision tree ensemble methods (RFR, ETR). Widely used implementations of scikit-learn (version 0.23.2)^11^ were employed for all ML models. For hyperparameter tuning, we tested a reasonable range of candidate values in an exhaustive way (grid search), as shown in Supplementary Table 3, we chose the best hyperparameter by 5-fold cross validation (CV) on the training set, and we used the model for calculating the predicted values for the test set (the hyperparameters not explicitly indicated in the table were set to the scikit-learn defaults). Namely, to avoid data leakage, we strictly followed a standard practice of "nested" CV, also known as double CV, to estimate the prediction accuracies; we used 5-fold CV for the internal CV and Monte Carlo CV (also known as repeated random subsampling CV) with 100 random leave-20%-out trials for the external CV, as explained in the main text.


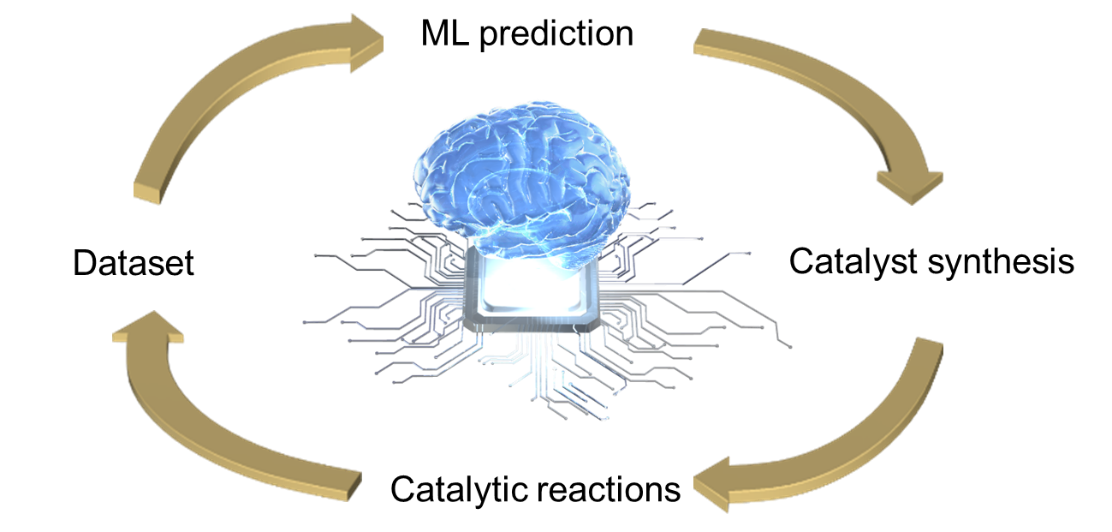


**Supplementary Fig. 1.** Schematics of the procedure of ML-assisted RWGS catalysts discovery.

**Supplementary Table 2.** Eight elemental properties (descriptors) used for ML predictions.

| Symbol | Group | EN_Allred | m.p. | d_fus_H | Density | BG_Oxide | Ox Num_Oxide | Eads_CO2 |
| --- | --- | --- | --- | --- | --- | --- | --- | --- |
| Li | 1 | 0.97 | 453.69 | 3.00 | 0.53 | 4.99 | 1 | -3.34 |
| Na | 1 | 1.01 | 370.87 | 2.60 | 0.97 | 2.04 | 1 | -0.86 |
| Mg | 2 | 1.23 | 923.00 | 8.48 | 1.74 | 4.64 | 2 | -1.12 |
| Al | 13 | 1.47 | 933.47 | 10.79 | 2.70 | 6.04 | 3 | -1.15 |
| Si | 14 | 1.74 | 1687.00 | 50.21 | 2.33 | 5.47 | 4 | -1.36 |
| K | 1 | 0.91 | 336.53 | 2.33 | 0.89 | 1.72 | 1 | -2.41 |
| Ca | 2 | 1.04 | 1115.00 | 8.54 | 1.54 | 3.69 | 2 | -4.22 |
| Sc | 3 | 1.20 | 1814.00 | 14.10 | 2.99 | 3.81 | 3 | -4.83 |
| Ti | 4 | 1.32 | 1941.00 | 14.15 | 4.51 | 2.06 | 4 | -4.90 |
| V | 5 | 1.45 | 2183.00 | 21.50 | 6.00 | 2.34 | 5 | -3.96 |
| Cr | 6 | 1.56 | 2180.00 | 21.00 | 7.15 | 2.43 | 3 | -2.15 |
| Mn | 7 | 1.60 | 1519.00 | 12.91 | 7.30 | 0.20 | 2 | -4.73 |
| Fe | 8 | 1.64 | 1811.00 | 13.81 | 7.87 | 0.00 | 3 | -1.07 |
| Co | 9 | 1.70 | 1768.00 | 16.06 | 8.86 | 0.00 | 2 | -0.78 |
| Ni | 10 | 1.75 | 1728.00 | 17.04 | 8.91 | 2.41 | 2 | -0.71 |
| Cu | 11 | 1.75 | 1357.77 | 12.93 | 8.96 | 0.51 | 1 | -1.05 |
| Zn | 12 | 1.66 | 692.68 | 7.07 | 7.14 | 0.73 | 2 | -0.73 |
| Ga | 13 | 1.82 | 302.91 | 5.58 | 5.91 | 2.04 | 3 | -0.87 |
| Ge | 14 | 2.02 | 1211.40 | 36.94 | 5.32 | 3.27 | 4 | -1.47 |
| Rb | 1 | 0.89 | 312.46 | 2.19 | 1.53 | 1.32 | 1 | -2.32 |
| Sr | 2 | 0.99 | 1050.00 | 7.43 | 2.64 | 3.45 | 2 | -3.80 |
| Y | 3 | 1.11 | 1799.00 | 11.40 | 4.47 | 4.11 | 3 | -3.26 |
| Zr | 4 | 1.22 | 2128.00 | 21.00 | 6.52 | 3.54 | 4 | -4.30 |
| Nb | 5 | 1.23 | 2750.00 | 30.00 | 8.57 | 1.68 | 5 | -2.65 |
| Mo | 6 | 1.30 | 2896.00 | 37.48 | 10.20 | 2.02 | 6 | -1.54 |
| Ag | 11 | 1.42 | 1234.93 | 11.28 | 10.50 | 0.06 | 1 | -1.01 |
| Cd | 12 | 1.46 | 594.22 | 6.21 | 8.69 | 0.00 | 2 | 0.14 |
| In | 13 | 1.49 | 429.75 | 3.28 | 7.31 | 0.96 | 3 | -1.55 |
| Sn | 14 | 1.72 | 505.08 | 7.17 | 7.26 | 1.41 | 4 | -1.00 |
| Sb | 15 | 1.82 | 903.78 | 19.79 | 6.68 | 3.41 | 3 | -0.92 |
| Cs | 1 | 0.86 | 301.59 | 2.09 | 1.93 | 0.62 | 1 | -2.22 |
| Ba | 2 | 0.97 | 1000.00 | 7.12 | 3.62 | 2.26 | 2 | -3.36 |
| La | 3 | 1.08 | 1193.00 | 6.20 | 6.15 | 3.88 | 3 | -4.12 |
| Ce | 3 | 1.08 | 1068.00 | 5.46 | 6.77 | 1.88 | 4 | -3.14 |
| Pr | 3 | 1.07 | 1208.00 | 6.89 | 6.77 | 3.88 | 3 | -3.79 |
| Nd | 3 | 1.07 | 1297.00 | 7.14 | 7.01 | 3.94 | 3 | -3.85 |
| Sm | 3 | 1.07 | 1345.00 | 8.62 | 7.52 | 3.88 | 3 | -3.90 |
| Eu | 3 | 1.01 | 1099.00 | 9.21 | 5.24 | 0.00 | 3 | -5.11 |
| Gd | 3 | 1.11 | 1585.00 | 10.00 | 7.90 | 3.13 | 3 | -4.13 |
| Tb | 3 | 1.10 | 1629.00 | 10.15 | 8.23 | 3.91 | 3 | -4.16 |
| Dy | 3 | 1.10 | 1680.00 | 11.06 | 8.55 | 3.94 | 3 | -4.53 |
| Ho | 3 | 1.10 | 1734.00 | 17.00 | 8.80 | 3.96 | 3 | -4.71 |
| Er | 3 | 1.11 | 1770.00 | 19.90 | 9.07 | 3.98 | 3 | -3.65 |
| Tm | 3 | 1.11 | 1818.00 | 16.84 | 9.32 | 3.99 | 3 | -4.99 |
| Yb | 3 | 1.06 | 1097.00 | 7.66 | 6.90 | 0.00 | 3 | -5.18 |
| Lu | 3 | 1.14 | 1925.00 | 22.00 | 9.84 | 4.03 | 3 | -5.13 |
| Hf | 4 | 1.23 | 2506.00 | 27.20 | 13.30 | 4.07 | 4 | -5.06 |
| W | 6 | 1.40 | 3695.00 | 52.31 | 19.30 | 1.57 | 6 | -2.06 |
| Re | 7 | 1.46 | 3459.00 | 60.43 | 20.80 | 2.68 | 7 | -0.51 |
| Bi | 15 | 1.67 | 544.40 | 11.15 | 9.79 | 2.25 | 3 | -0.89 |

The electronegativity according to the Allred-Rochow definition (EN_Allred) was obtained from an original report by Allred and Rochow.^12^ Melting point in K (m.p.), enthalpy of formation (d_fus_H), and density at 25 °C in g cm^-3^ were obtained from the *CRC Handbook of Chemistry and Physics*.^13^ The BG in the most stable oxide from (BG Oxide) and oxidation number in the most stable oxide form (Ox Num_Oxide) were obtained from the Materials Project. Adsorption energy of CO_2_ (Eads_CO2) in eV was obtained by DFT calculations using VASP (See the Methods section for the detailed calculation conditions).


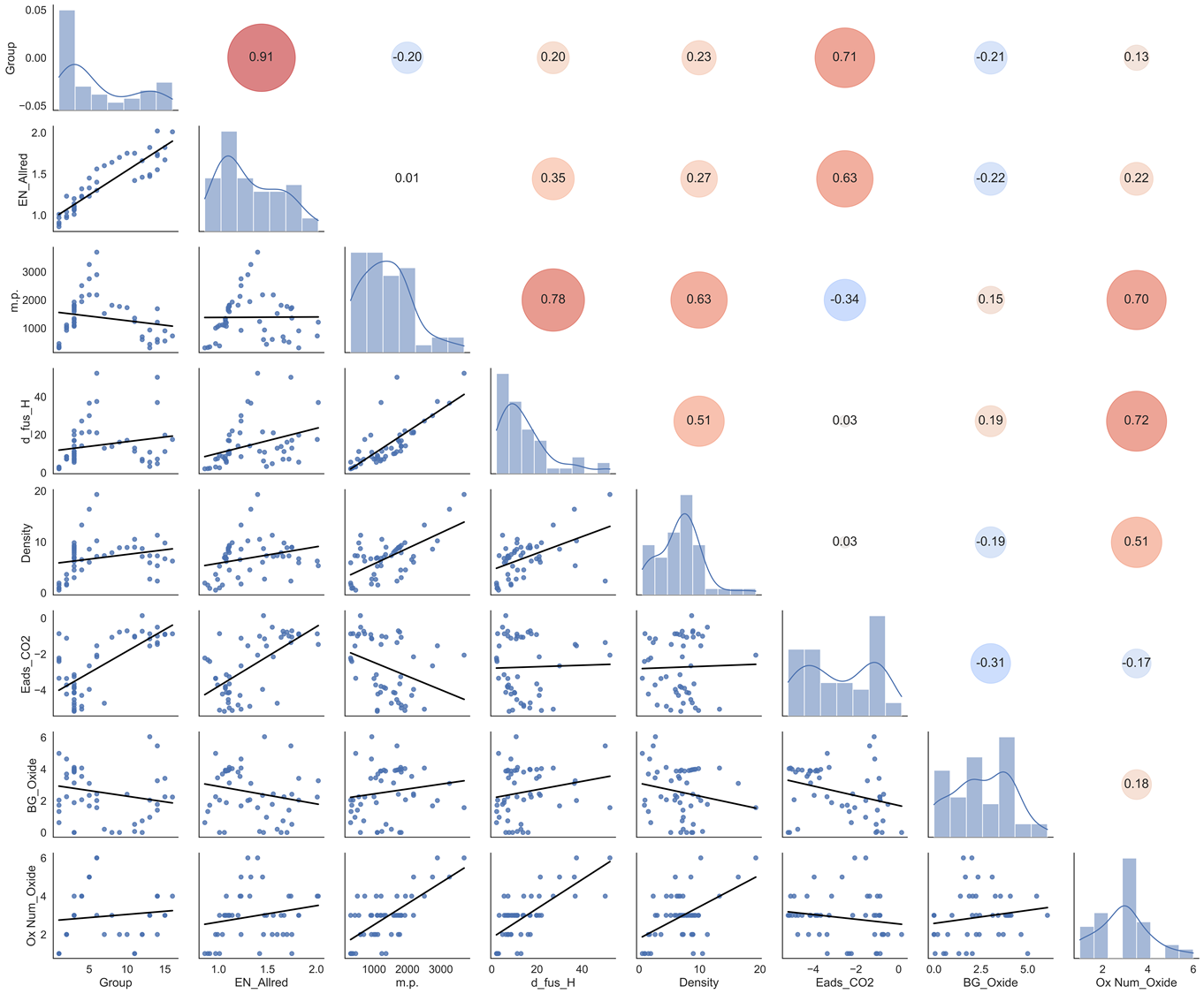


**Supplementary Fig. 2.** Correlation map for the eight selected descriptors.

**Supplementary Table 3.** Ranges used in the hyperparameter searches for the ML methods (if not specified, the default values of scikit-learn were used).

| Type | | Method | Hyperparameters [Tested range] |
| --- | --- | --- | --- |
| Linear | | Lasso | alpha ∈ [10^-2^,10^-1^,1.0,10^1^,10^2^] |
|  |  | Ridge | alpha ∈ [10^-2^,10^-1^,1.0,10^1^,10^2^] |
| Nonlinear | Kernel methods | GPR | kernel=Matern 5/2, alpha = 0.01,  n_restarts_optimizer = 10,  normalize_y = True |
|  |  | SVR | kernel=’rbf’, C ∈ [1.0,10,10^2^,…, 10^5^], gamma ∈ [1.0,10^-1^,10^-2^,…,10^-9^,10^-10^], epsilon ∈ [10^-2^,10^-1^,1.0,10^1^,10^2^] |
|  | Tree　ensemble  methods | RFR | n_estimators ∈ [100, 250, 500, 1000, 1500], max_depth ∈ [3, 4, 5,…, 9] |
|  |  | ETR | n_estimators ∈ [100, 250, 500, 1000, 1500], max_depth ∈ [3, 4, 5,…, 9] |

**
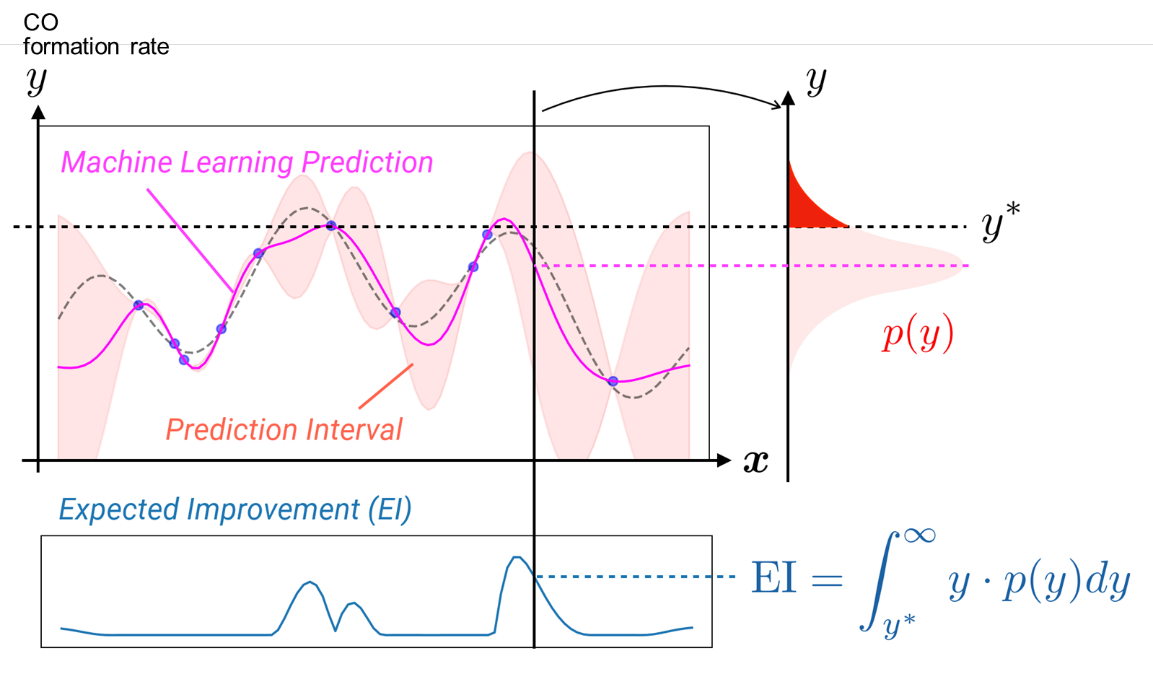
**

**Supplementary Fig. 3.** Description of the expected improvement (EI). The EI score $\mathbb{E}\left\{ \text{max}\left( \mu\left( \boldsymbol{x} \right)-y^{*},0 \right) \right\}$ corresponds to the average of the improvement in $y$ (CO formation rate) weighted by the probability of the red-colored region, thus considering not only the predicted values but also their predicted *variances*. Because the ML prediction (pink curve) usually cannot exceed the best (maximum) value of the training data by definition, it is inadequate to directly use the predicted values to attempt to find data points with higher CO formation rates.


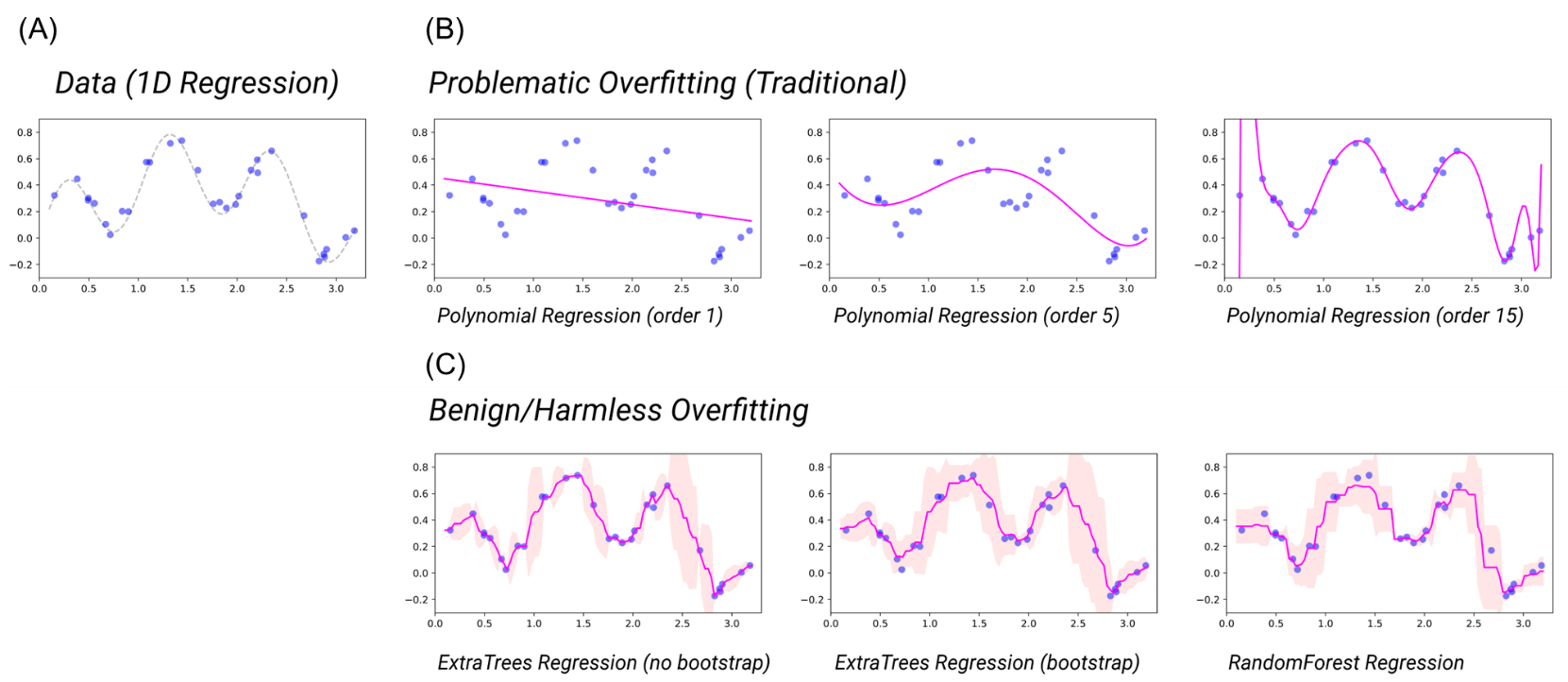


**Supplementary Fig. 4.** Intuitive example to understand why overfitting to training data does not necessarily imply an increase in the test error in modern ML and thus why near-zero training (almost perfect fitting) can work in noisy cases. (A) A toy regression example. Blue points are samples from the function (dashed line) by adding random noise. (B) Examples of polynomial regression. In this traditional case, if we increase the number of model parameters, we observed very problematic overfitting to the training samples. Polynomial regression (order p) corresponds to polynomial regression with degree p. For example, p = 1 is a linear regression. For p = 15, we observed near-zero training errors (w.r.t RMSE and R^2^ for the training data), but the fitted curve clearly does not make sense. (C) Results of our choice of ML models. For these models, we observe that the fitted curves somehow capture the underlying unknown function (dashed line) even when we still observe near-zero training errors (almost perfectly fitting the training data). This is a toy 1D regression to give an intuitive understanding, but modern ML usually handles higher-dimensional cases, and thus, these effects are clearer. Given a small number of data points (a statistically underspecified situation), interpolating noisy training data as is can be informative. This example also contrasts the difference between ETR (pseudo-piecewise linear) and RFR (stepwise). This difference has more influence in the high-dimensional cases handled in this paper. Prediction intervals (confidence intervals) for evaluating the uncertainty levels are also shown with a light red color.


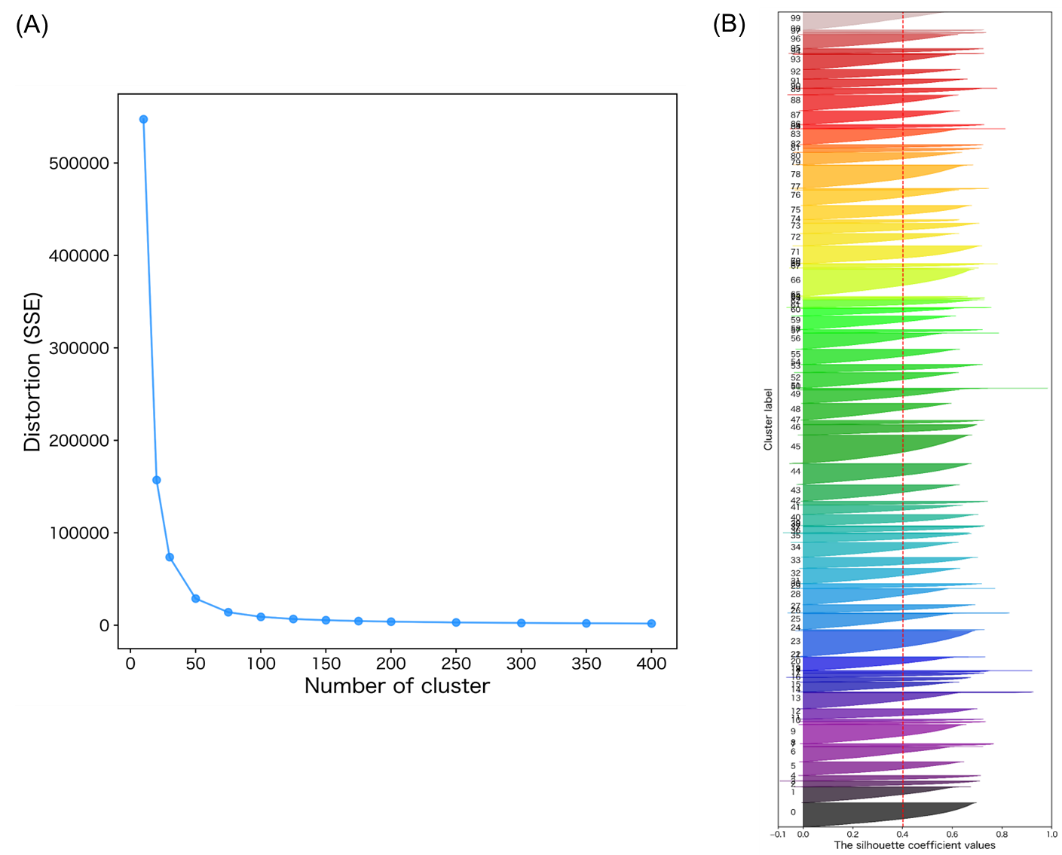


**Supplementary Fig. 5.** Representative (A) elbow and (B) silhouette analyses used to assess the optimal number of clusters (K) using 300 datapoints (See the data directory in the GitHub repository https://github.com/shinya-mine) with explorative ML methods based on ETR. In (A), “SSE” means the sum of squared errors of prediction.

Supplementary Results

**Supplementary Table 4.** Reports on catalytic RWGS reactions (225 °C ≤ *T* ≤ 250 °C).

| Catalyst | H_2_/CO_2_ | *T* (°C) | *P* (bar) | CO_2_ conv. (%) | CO selec. (%) | CO form. rate 　　　　(mmol g_cat_^-1^ min^-1^) | Reference |
| --- | --- | --- | --- | --- | --- | --- | --- |
| Pt(2)/CeO_2_ | 1 | 225 | 1 | 13.7 | 99.0 | 0.07 | ^14^ |
| Ni_12_P_5_(10.4)/SiO_2_ (Light) *^a^* | 5 | 240 | 1.2 | 0.04 | 99.7 | 0.081 | ^15^ |
| Ni_12_P_5_(10.4)/SiO_2_ (Dark) | 5 | 240 | 1.2 | 0.01 | 99.7 | 0.020 |  |
| Cu(10)-C/ZnO(5)/SiO_2_ | 3 | 250 | 30 | 6.0 | 99.0 | <0.05 | ^16^ |
| Li(10)-Rh(5)/Zeolite | 3 | 250 | 30 | 13.0 | 87.0 | 0.12 | ^17^ |
| Rh(0.45)@S-1 | 3 | 250 | 10 | 3.2 | 93.1 | 0.015 | ^18^ |
| K(0.8)-Pt(0.05)/zeolite L | 1 | 250 | 1 | 1.0 | 100 | 0.19 | ^19^ |
| Ni(2.4)/SiO_2_ | 4 | 250 | 1 | 3.2 | 100 | 0.18 | ^20^ |
| CuMgAl-LDH (Cu/Mg/Al=0.3/1.7/1) | 4 | 250 | 1 | 4.5 | 100 | 0.14 | ^21^ |
| Au(1)/TiO_2_ | 4 | 250 | 1 | 11.5 | 100 | 0.13 | ^22^ |
| Pt(5)/TiO_2_ | 1 | 250 | 1 | 4.0 | 97.5 | 0.07 | ^23^ |
| NH_2_MPA/Pt(5)/TiO_2_ | 14 | 250 | 1 | 8.4 | 95.1 | 0.06 | ^24^ |
| Pt(1)/CeO_2_ | 1 | 250 | 1 | 0.6 | 100 | 0.06 | ^25^ |
| In_2_O_3_(50)-CeO_2_ | 1 | 250 | 1 | <0.1 | - | <0.05 | ^26^ |
| Pd(3)In(8)/SiO_2_ | 1 | 250 | 1 | <0.1 | - | <0.05 | ^27^ |
| H-SiO_2_@Ru(0.48)@SiO_2_ | 1 | 250 | 1 | 0.1 | 99.5 | 0.015 | ^28^ |
| Au(2.76)/TiO_2_ | 4 | 250 | 1 | 1.5 | 100 | 0.064 | ^29^ |
| Pt(2)/CeO_2_ | 12.5 | 250 | 1 | 29.8 | 100 | 0.497 | ^30^ |
| Cu(0.5)Ni(0.5)/SiO_2_ | 4 | 250 | 1 | 4.0 | 100 | 0.008 | ^31^ |
| Rh(0.05)/Fe_3_O_4_ | 4 | 250 | 1 | 0.9 | 93.8 | 0.191 | ^32^ |
| Pt(0.8)/CeO_2_ | 3 | 250 | 1 | 3.0 | 100 | 0.223 | ^33^ |
| Ru(1.9)/MoO_3_ | 3 | 250 | 1 | 8.3 | 97.2 | 0.017 | ^34^ |
| Pt(2)-MoO_x_/Mo_2_N | 3 | 250 | 1 | 11.4 | 100 | 5.46 | ^35^ |
| Pd(1)Cu(0.2)/WO_3_ (Light) *^a^* | 1 | 250 | 1 | 0.02 | 100 | 0.020 | ^36^ |
| Pd(1)Cu(0.2)/WO_3_ (Dark) | 1 | 250 | 1 | 0.01 | 100 | 0.011 |  |
| Pd(1)Ba(0.2)/WO_3_ (Light) *^a^* | 1 | 250 | 1 | 0.05 | 100 | 0.042 | ^37^ |
| Pd(1)Ba(0.2)/WO_3_ (Dark) | 1 | 250 | 1 | 0.03 | 100 | 0.021 |  |
| Pd(1)@WO_3_ (Light) *^a^* | 1 | 250 | 1 | N/A | 99 | 0.05 | ^38^ |
| Pd(1)@WO_3_ (Dark) | 1 | 250 | 1 | N/A | 99 | 0.03 |  |
| In_2_O_3_ (Light) *^a^* | 1 | 250 | 1 | - | 87.9 | 0.0017 | ^39^ |
| In_2_O_3_ (Dark) | 1 | 250 | 1 | - | 87.9 | 0.0002 |  |
| Pt(3)/Mo(10)/TiO_2_ *^b^* | 3 | 250 | 1 | 3.1 | 100 | 2.55 | *Our previous study^40^ |
| Pt(3)/Rb(1)-Ba(1)-Mo(0.6)-Nb(0.2)/TiO_2_ | 3 | 250 | 1 | 4.9 | 100 | 4.10 | This study^41^ |
| Pt(3)/Rb(1)-Ba(1)-Mo(0.6)-Nb(0.2)/TiO_2_*^c^* | 3 | 250 | 1 | 1.9 | 100 | 6.28 |  |

*^a^* Photothermal RWGS reaction *^b^* The activity was tested under the reaction conditions applied in this study. *^c^* The reaction was performed using a higher space velocity (5 mg of catalyst, 40 mL min^-1^ CO_2_, 120 mL min^-1^ H_2_, and 1 mL min^-1^ N_2_).

**Supplementary Table 5.** Catalytic RWGS performance of Pt(3)/Rb(1)-Ba(1)-Mo(0.6)-Nb(0.2)/TiO_2_ and reference catalysts for comparison. Reaction conditions: 20 mL min^-1^ CO_2_, 60 mL min^-1^ H_2_, 5 mL min^-1^ N_2_, and 250 °C.

| ID | Catalyst | CO yield (%) | CO selec. (%) | CO form. Rate　(mmol g_cat_^-1^ min^-1^) |
| --- | --- | --- | --- | --- |
| 191 | Pt(3)/Rb(1)-Ba(1)-Mo(0.6)-Nb(0.2)/TiO_2_ | 4.91 | 100 | 4.10 |
| 301 | Rb(1)-Ba(1)-Mo(0.6)-Nb(0.2)/TiO_2_ | 0 | - | 0 |
| 302 | Pt(3)/Rb(1)-Ba(1)-Mo(0.6)/TiO_2_ | 4.21 | 100 | 3.51 |
| 303 | Pt(3)/Rb(1)-Ba(1)-Nb(0.2)/TiO_2_ | 2.68 | 100 | 2.23 |
| 304 | Pt(3)/Rb(1)-Mo(0.6)-Nb(0.2)/TiO_2_ | 4.28 | 100 | 3.56 |
| 305 | Pt(3)/Ba(1)-Mo(0.6)-Nb(0.2)/TiO_2_ | 4.61 | 100 | 3.84 |
| 306 | Pt(3)/Ba(1)-Nb(0.2)/TiO_2_ | 2.21 | 100 | 1.84 |
| 307 | Pt(3)/Mo(0.6)-Nb(0.2)/TiO_2_ | 3.74 | 100 | 3.11 |
| 308 | Pt(3)/Rb(1)-Ba(1)/TiO_2_ | 1.98 | 100 | 1.65 |
| 309 | Pt(3)/Rb(1)-Nb(0.2)/TiO_2_ | 1.74 | 100 | 1.45 |
| 310 | Pt(3)/Rb(1)-Mo(0.6)/TiO_2_ | 4.05 | 100 | 3.04 |
| 311 | Pt(3)/Ba(1)-Mo(0.6)/TiO_2_ | 3.64 | 100 | 3.03 |
| 312 | Pt(3)/Rb(1)/TiO_2_ | 1.88 | 100 | 1.57 |
| 313 | Pt(3)/Nb(0.2)/TiO_2_ | 1.98 | 100 | 1.62 |
| 314 | Pt(3)/Mo(0.6)/TiO_2_ | 4.18 | 100 | 3.48 |
| 315 | Pt(3)/Ba(1)/TiO_2_ | 2.16 | 100 | 1.80 |
| 316 | Pt(3)/TiO_2_ | 1.87 | 100 | 1.56 |
| 317 | Pt(3)/Rb(1)-Ba(1)-Mo(0.6)-Nb(0.2)/SiO_2_ | 3.60 | 100 | 3.00 |
| 318 | Pt(3)/Rb(1)-Ba(1)-Mo(0.6)-Nb(0.2)/γ-Al_2_O_3_ | 1.08 | 100 | 0.90 |
| 319 | Pt(3)/Rb(1)-Ba(1)-Mo(0.6)-Nb(0.2)/ZrO_2_ | 1.46 | 100 | 1.22 |
| 320 | Pt(3)/Rb(1)-Ba(1)-Mo(0.6)-Nb(0.2)/CeO_2_ | 2.09 | 100 | 1.73 |
| 321 | Pt(3)/Rb(1)-Ba(1)-Mo(0.6)-Nb(0.2)/MgO | 1.17 | 100 | 0.98 |
| 322 | Pt(3)/Rb(1)-Ba(1)-Mo(0.6)-Nb(0.2)/Nb_2_O_5_ | 3.42 | 100 | 2.85 |
| 323 | Pt(3)/Rb(1)-Ba(1)-Mo(0.6)-Nb(0.2)/TiO_2_(ST01) | 2.64 | 100 | 2.20 |
| 324 | Pt(3)/Rb(1)-Ba(1)-Mo(0.6)-Nb(0.2)/TiO_2_(STR100N) | 3.64 | 100 | 3.03 |
| 325 | Pt(3)/Rb(1)-Ba(1)-Mo(0.6)-Nb(0.2)/Carbon | 0.89 | 100 | 0.74 |
| 326 | Re(3)/Rb(1)-Ba(1)-Mo(0.6)-Nb(0.2)/TiO_2_ | 0.28 | 100 | 0.24 |
| 327 | Ru(3)/Rb(1)-Ba(1)-Mo(0.6)-Nb(0.2)/TiO_2_ | 0.96 | 100 | 0.80 |
| 328 | Ir(3)/Rb(1)-Ba(1)-Mo(0.6)-Nb(0.2)/TiO_2_ | 1.03 | 100 | 0.86 |
| 329 | Rh(3)/Rb(1)-Ba(1)-Mo(0.6)-Nb(0.2)/TiO_2_ | 2.28 | 100 | 1.91 |
| 330 | Pd(3)/Rb(1)-Ba(1)-Mo(0.6)-Nb(0.2)/TiO_2_ | 2.35 | 100 | 1.96 |
| 331 | Ag(3)/Rb(1)-Ba(1)-Mo(0.6)-Nb(0.2)/TiO_2_ | 0.11 | 100 | 0.09 |
| 332 | Cu/ZnO/Al_2_O_3_ (HiFUEL® W220) | 0.98 | 100 | 0.82 |
| 333 | FeCrCuO*_x_* (HiFUEL® W210) | 0.68 | 100 | 0.56 |





**Supplementary Fig. 6.** Comparison of prediction accuracy (RMSE, MSE, MAE, and R^2^) at each iteration for the CO formation rate of the RWGS catalysts using ML models based on three types of descriptor representation.


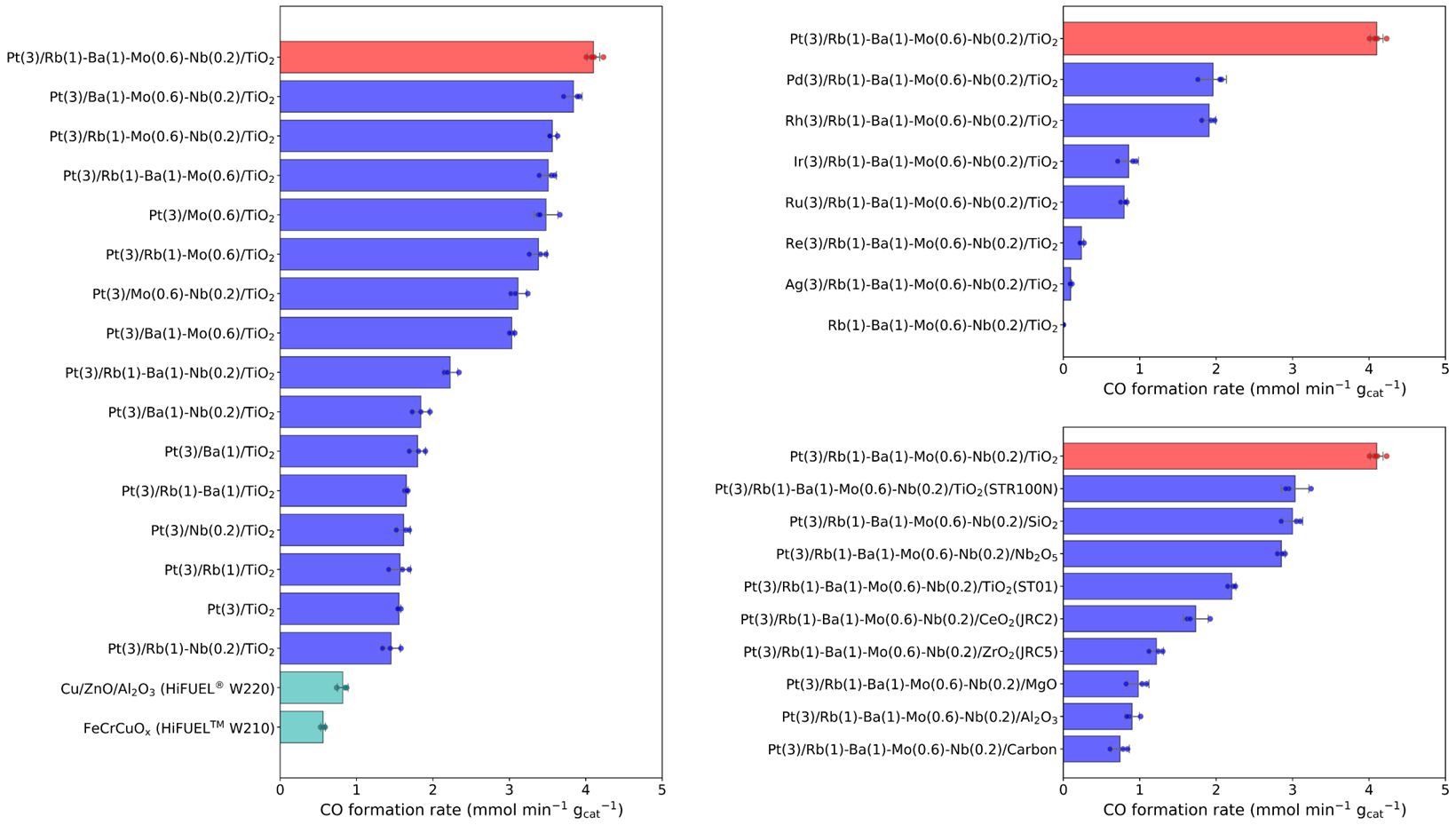


**Supplementary Fig. 7.** Catalytic RWGS performance of Pt(3)/Rb(1)-Ba(1)-Mo(0.6)-Nb(0.2)/TiO_2_ and reference catalysts for comparison. Effects of (A) additive oxide, (B) active metal, and (C) support. Error bars represent the standard deviation.

**Supplementary Text 1 (Exploratory Data Analysis)**

The correlations between CO formation rates and the values of each descriptor for the 300 RWGS catalysts are visualized in Supplementary Fig. 10. Among the eight descriptors employed in this study, EN was most strongly correlated with the CO formation rate. However, its R^2^ value was low (0.26), reflecting the complex and non-linear nature of the catalytic reaction and clearly showing that the catalytic activity (RWGS activity) cannot be described with a single descriptor.


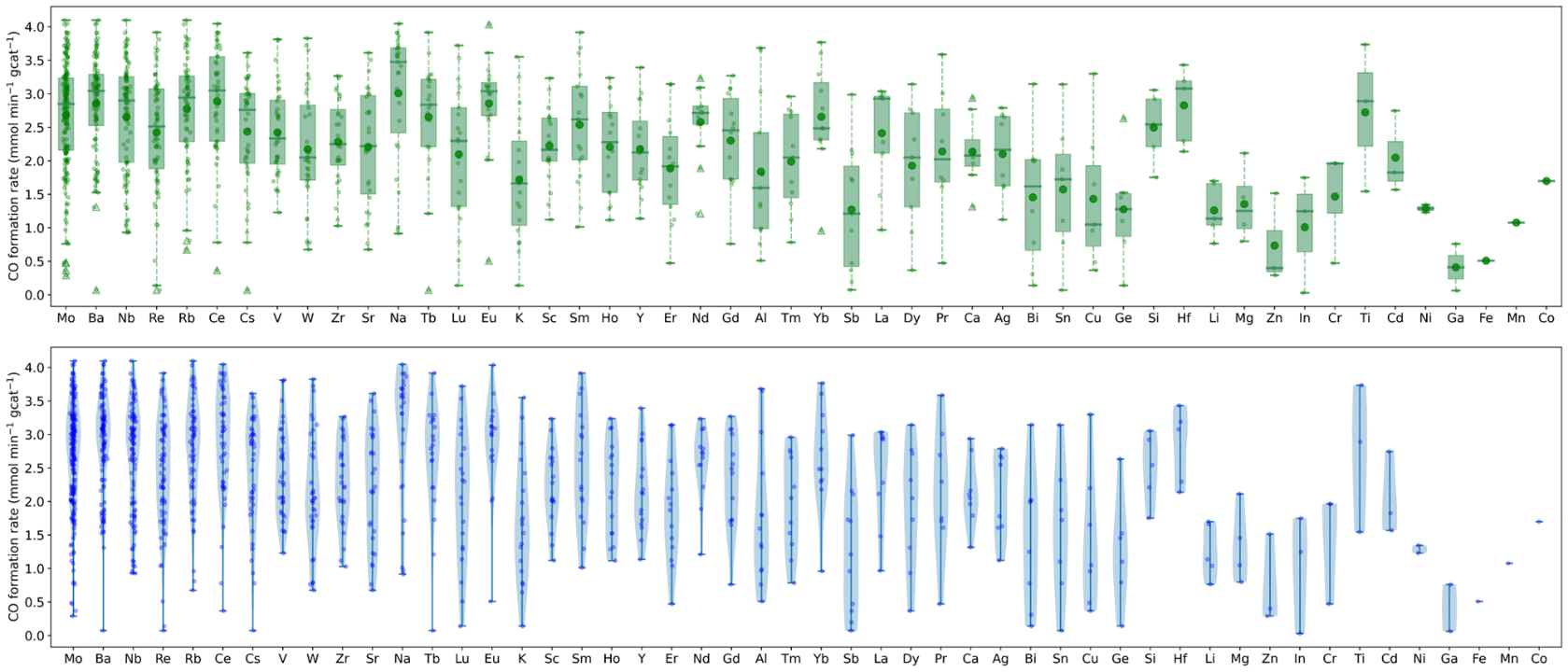


**Supplementary Fig. 8.** Visualization of RWGS catalyst datasets. Boxplot for each additive oxide component. The boxes represent the interquartile range, spanning from the first quartile to the third quartile, with a horizontal line indicating the median. Whiskers extend up to 1.5 times the interquartile range, encompassing the largest and smallest observed data points falling within this range. Individual data points are displayed as dots.


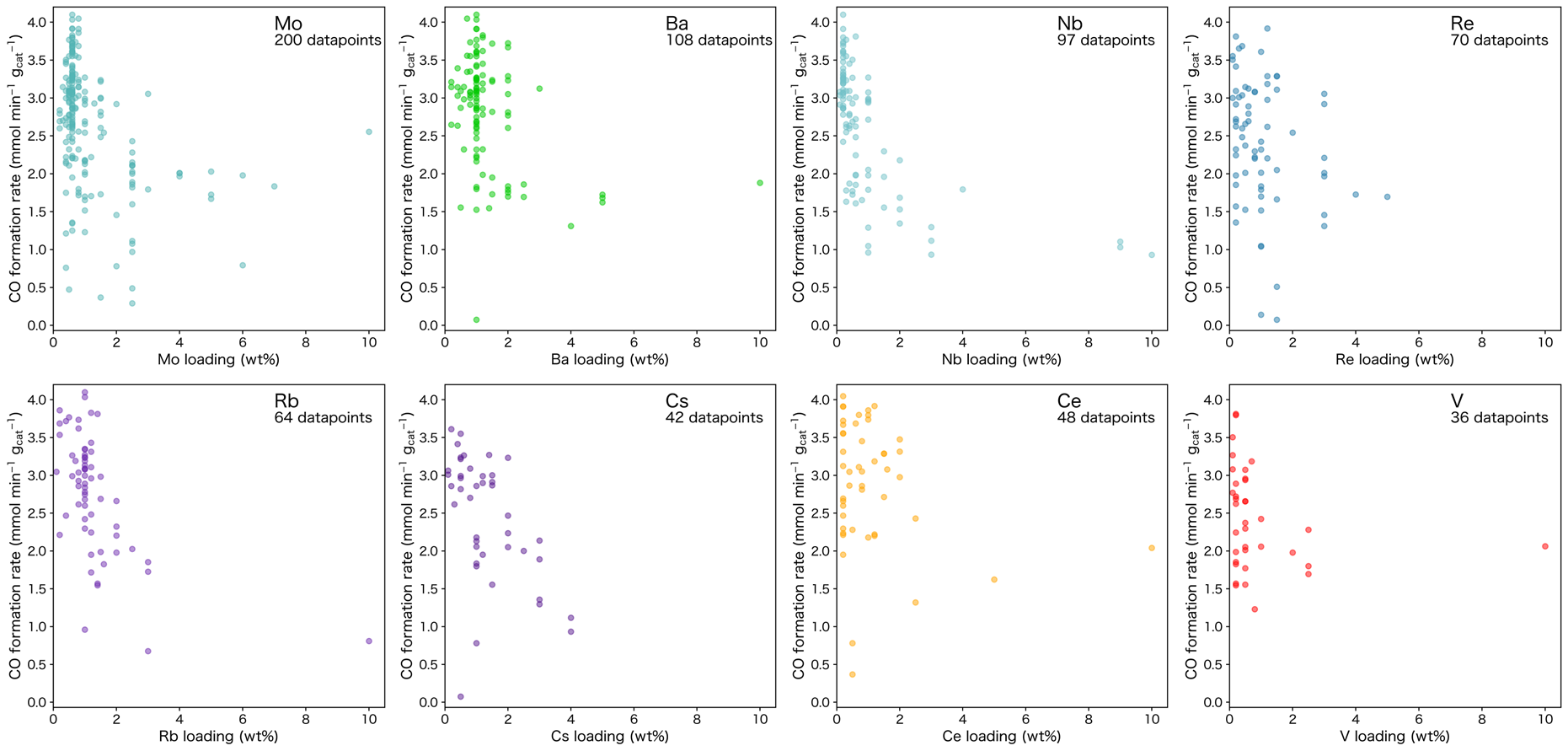


**Supplementary Fig. 9.** Effects of the loading amount of Mo, Ba, Nb, Re, Rb, Ce, Cs, and V on the CO formation rate in the RWGS reaction.


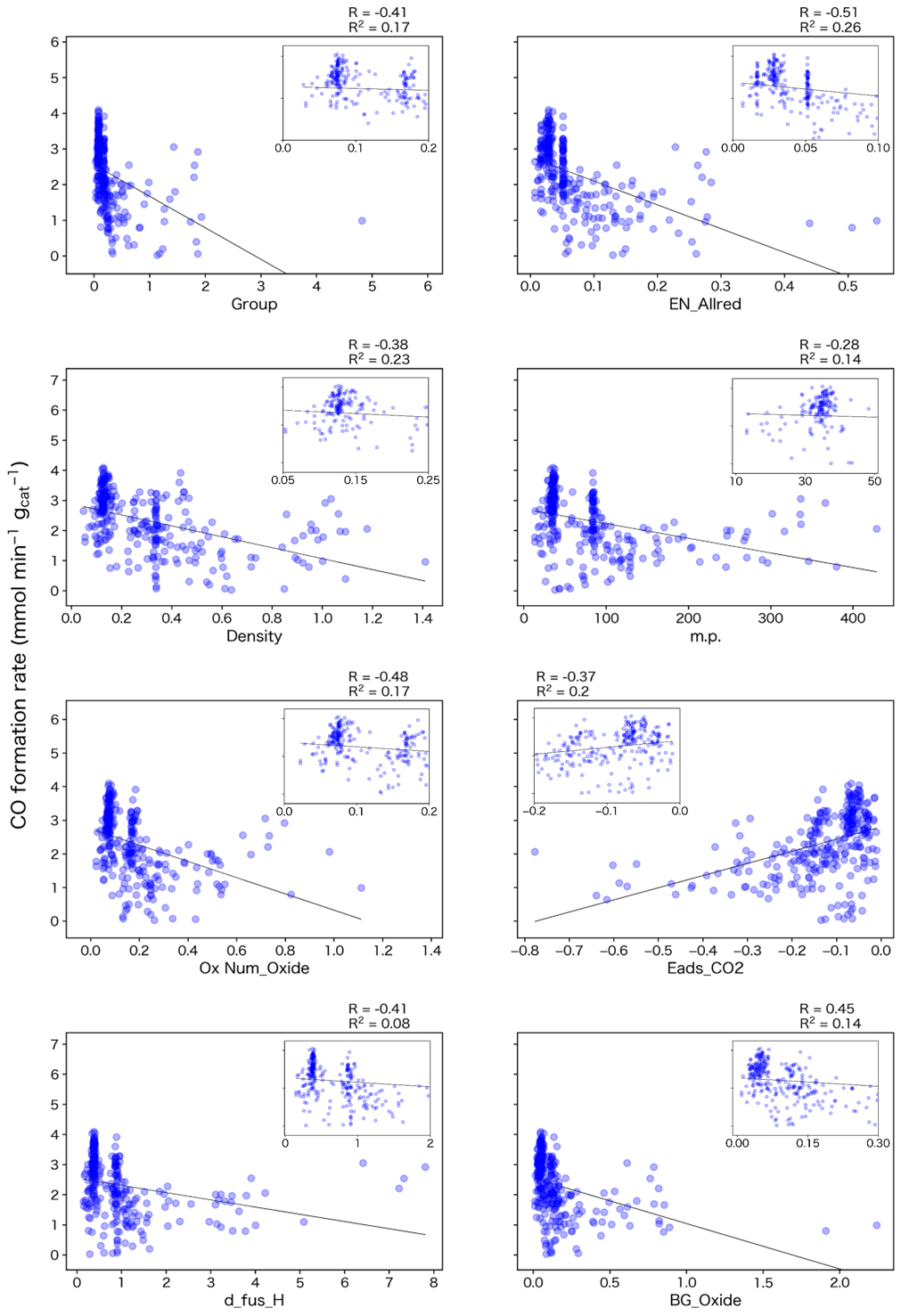


**Supplementary Fig. 10.** Correlation between the CO formation rates and each descriptor value for the 300 RWGS catalysts. The regions near the zero values are enlarged in the insets.

**Supplementary Text 2 (ML Analysis Using Explorative Elemental Descriptor Representation)**

The prediction accuracy was quantified based on four criteria (R^2^, MAE, RMSE, and MSE) by 10-fold cross validation, as shown in Supplementary Fig. 11. Among the six tested ML models, ETR gave the highest predictive accuracy for all of the criteria. Supplementary Fig. 12 provides 90%/10% training-test error plots and 100% test error plots for the best model using ETR. Although EN_Allred and group are highly correlated (as shown in Supplementary Fig. 2), they were both found to be individually important in predicting the CO formation rate, as the prediction results without EN_Allred (RSME = 0.42, test R^2^ value = 0.79) and without group (RSME = 0.43, test R^2^ value = 0.78) were inferior to those obtained with the optimal combination of the eight descriptors (RSME = 0.40, test R^2^ value = 0.81).


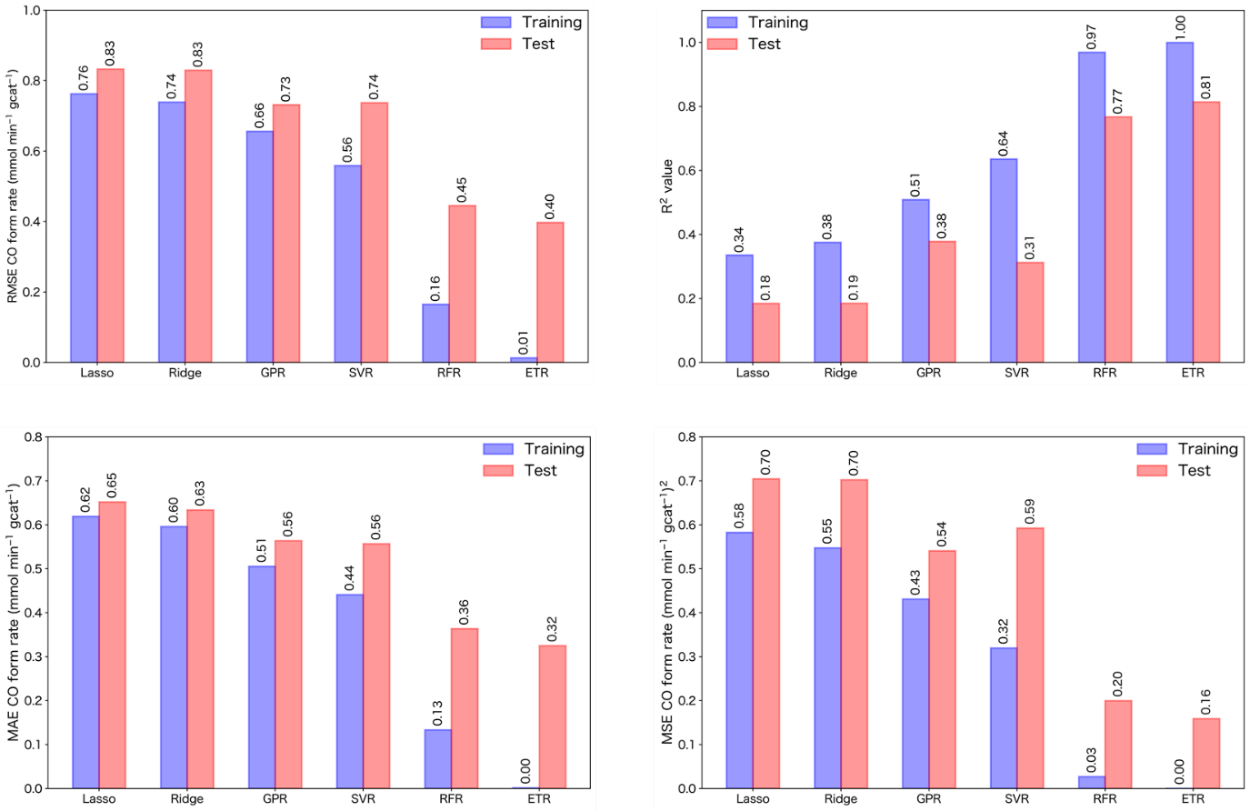


**Supplementary Fig. 11.** Comparison of the prediction accuracy for the CO formation rate of the 300 RWGS catalysts using ML models based on the explorative elemental descriptor representation.


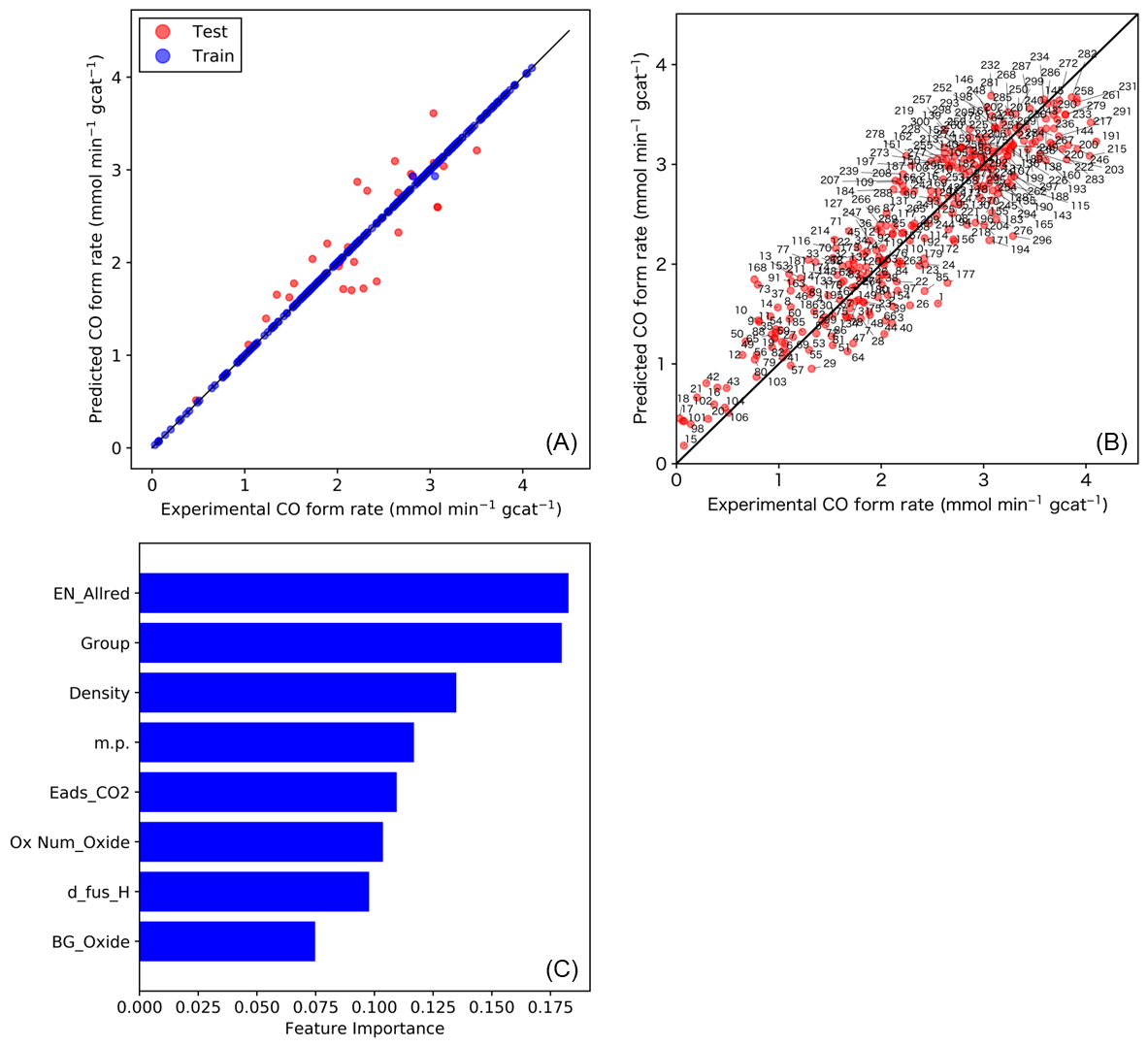


**Supplementary Fig. 12.** Experimental and predicted values of CO formation rates. Cross validation was performed using ETR combined with explorative elemental descriptor representation. (A) 90%/10% training-test error plots and (B) 100% test error plots. Blue points represent training data, while red points represent test data. Numbers in the right Fig. correspond to the catalyst ID.

**
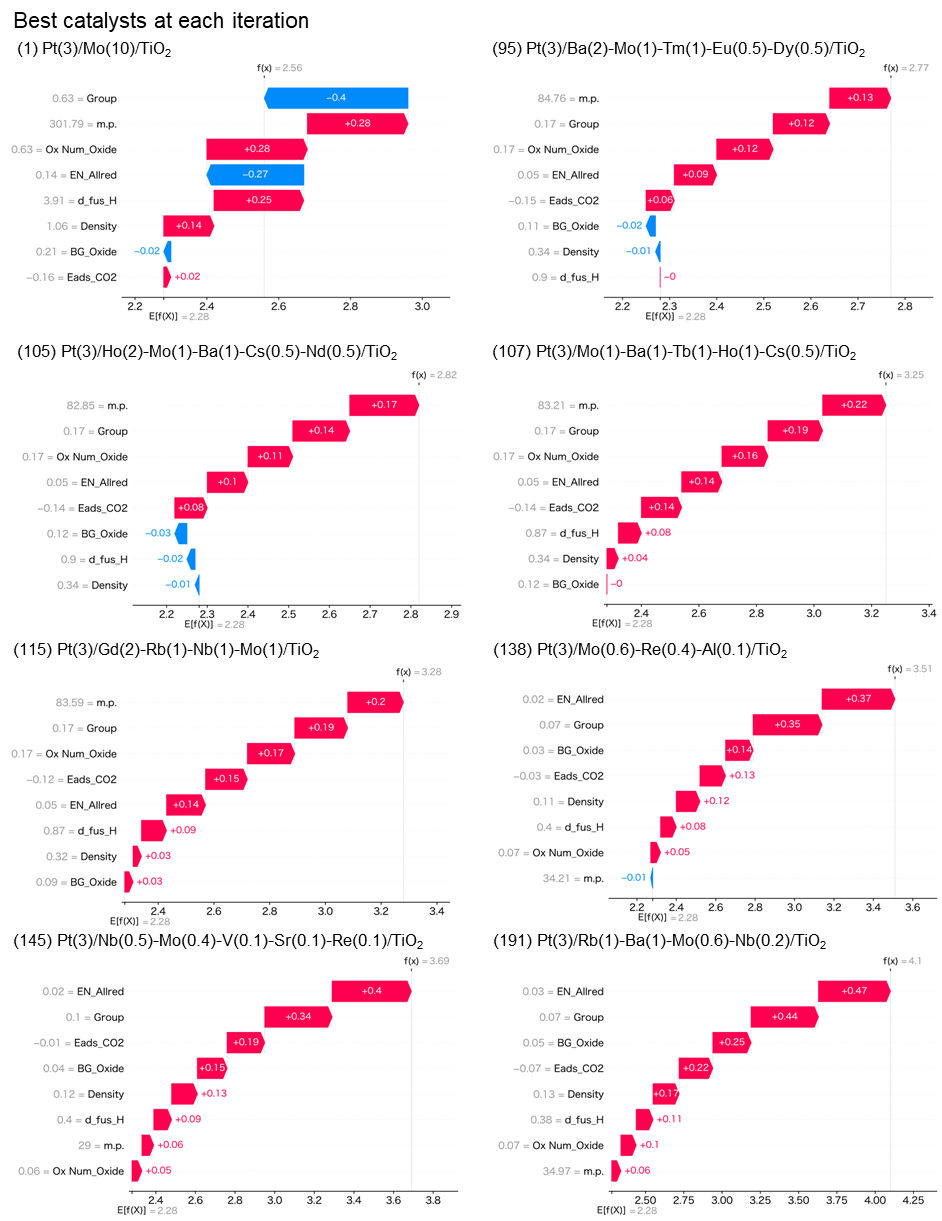
**

**Supplementary Fig. 13.** Breakdown of Shapley Additive exPlanations (SHAP) values as waterfall plots for the eight catalysts featured in Fig. 1 in the main text to determine which feature values are responsible for increases and decreases from the base value (dataset average: E[f(X)]) of 2.28 relative to the predicted value. Positive and negative contributions of each feature (SHAP values: f(x)) are shown in red and blue, respectively. The explorative elemental descriptor representation was used.


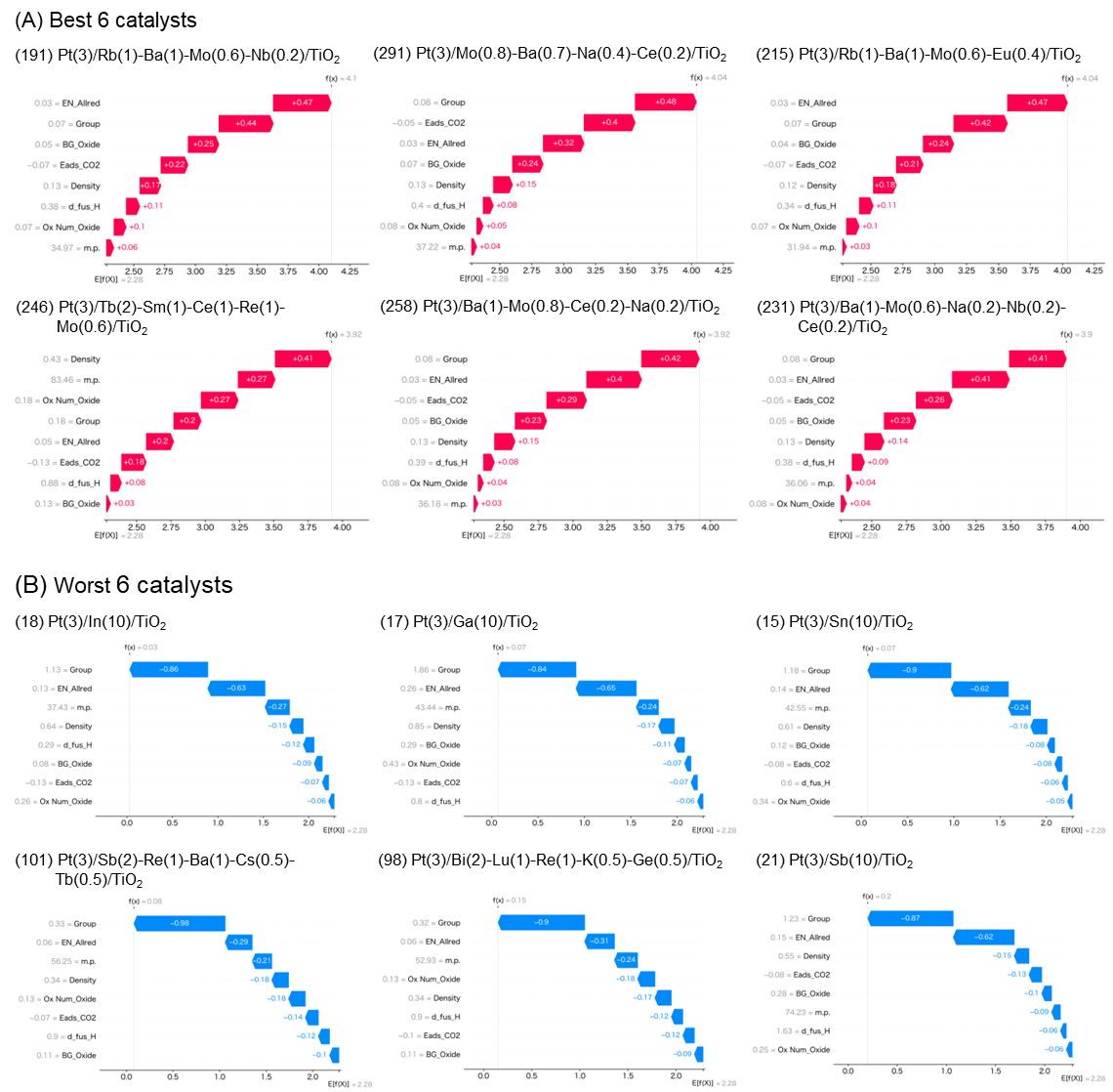


**Supplementary Fig. 14.** Breakdown of SHAP values as waterfall plots for the (A) best six catalysts and (B) worst six catalysts to determine which feature values are responsible for increases and decreases from the base value (dataset average: E[f(X)]) of 2.28 relative to the predicted value. Positive and negative contributions of each feature (SHAP values: f(x)) are shown in red and blue, respectively. The explorative elemental descriptor representation was used.

**Supplementary Text 3 (ML Analysis Using Exploitative Elemental Descriptor Representation)**

A ML analysis using exploitative elemental descriptor representation was also performed, as shown in Supplementary Figs. 15–18. As this method directly considers the elemental composition in addition to the elemental descriptors and shows relatively high prediction accuracy, this analysis makes it easier to understand the contributing elements in the given data. Similar to the ML analysis using the explorative elemental descriptor representation, ETR provided the most accurate prediction in all the criteria among the six ML models tested. In addition, feature importance and SHAP analyses showed similar results, again confirming the high reliability of the analysis.

Analysis of the SHAP values using waterfall plots for the eight catalysts showed the best CO formation rate at each iteration (Supplementary Fig. 17), and the best six catalysts (Supplementary Fig. 18A) and worst six catalysts (Supplementary Fig. 18B) were obtained. This analysis reveals the descriptors that are responsible for the increases or decreases from the dataset average value (2.28) relative to the predicted value. For instance, elements such as Mo, Na, Ba, and Tb were found to be highly influential and positively affect the catalytic performance, whereas elements such as Sb and Bi have negative impacts.


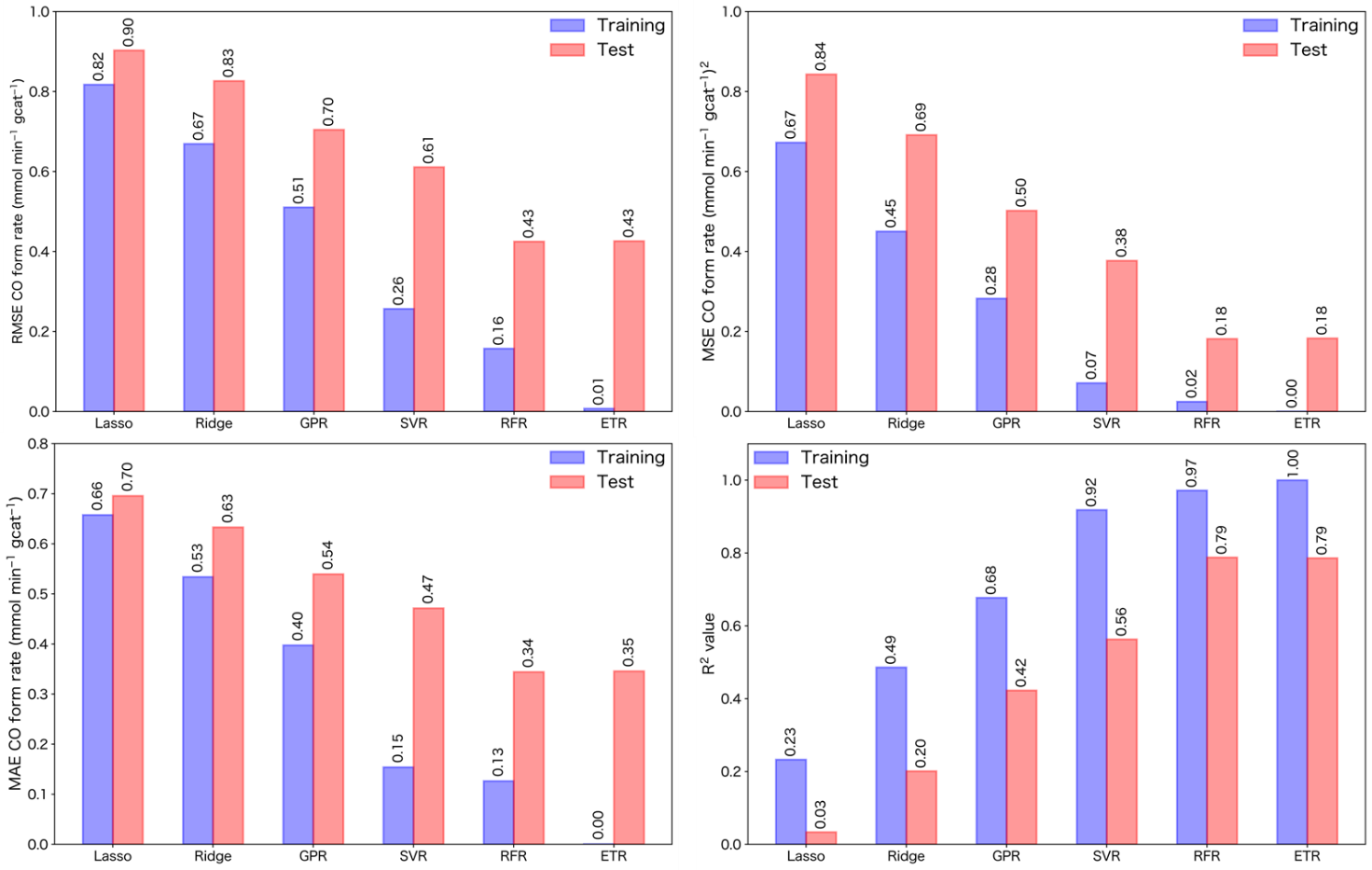


**Supplementary Fig. 15.** Comparison of the prediction accuracy for the CO formation rate using ML models based on the exploitative elemental descriptor representation.


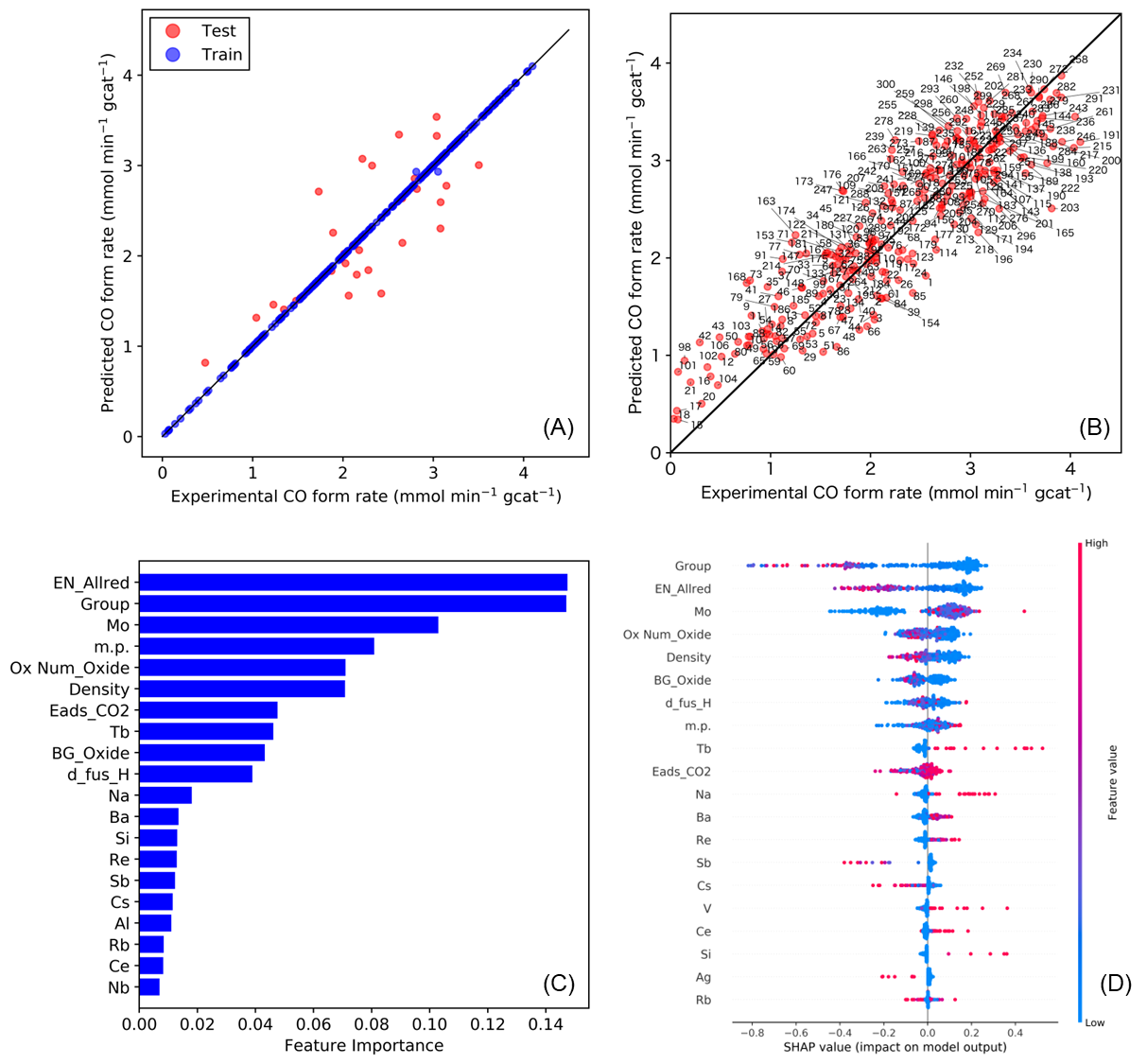


**Supplementary Fig. 16.** Experimental and predicted values of CO formation rates. Cross validation was performed using ETR combined with the exploitative elemental descriptor representation. (A) 90%/10% training-test error plots and (B) 100% test error plots. Blue points represent training data, while red points represent test data. Numbers in the right Fig. correspond to the catalyst ID. (C) Feature-importance and (D) SHAP values of the descriptors used to predict CO formation rates. The SHAP values of individual factors are plotted as dots (blue and red correspond to low and high features, respectively). Here, features appear in descending order according to the sum of their absolute SHAP values. Dots are displaced vertically to visualize the density of data points at a given SHAP value.

**
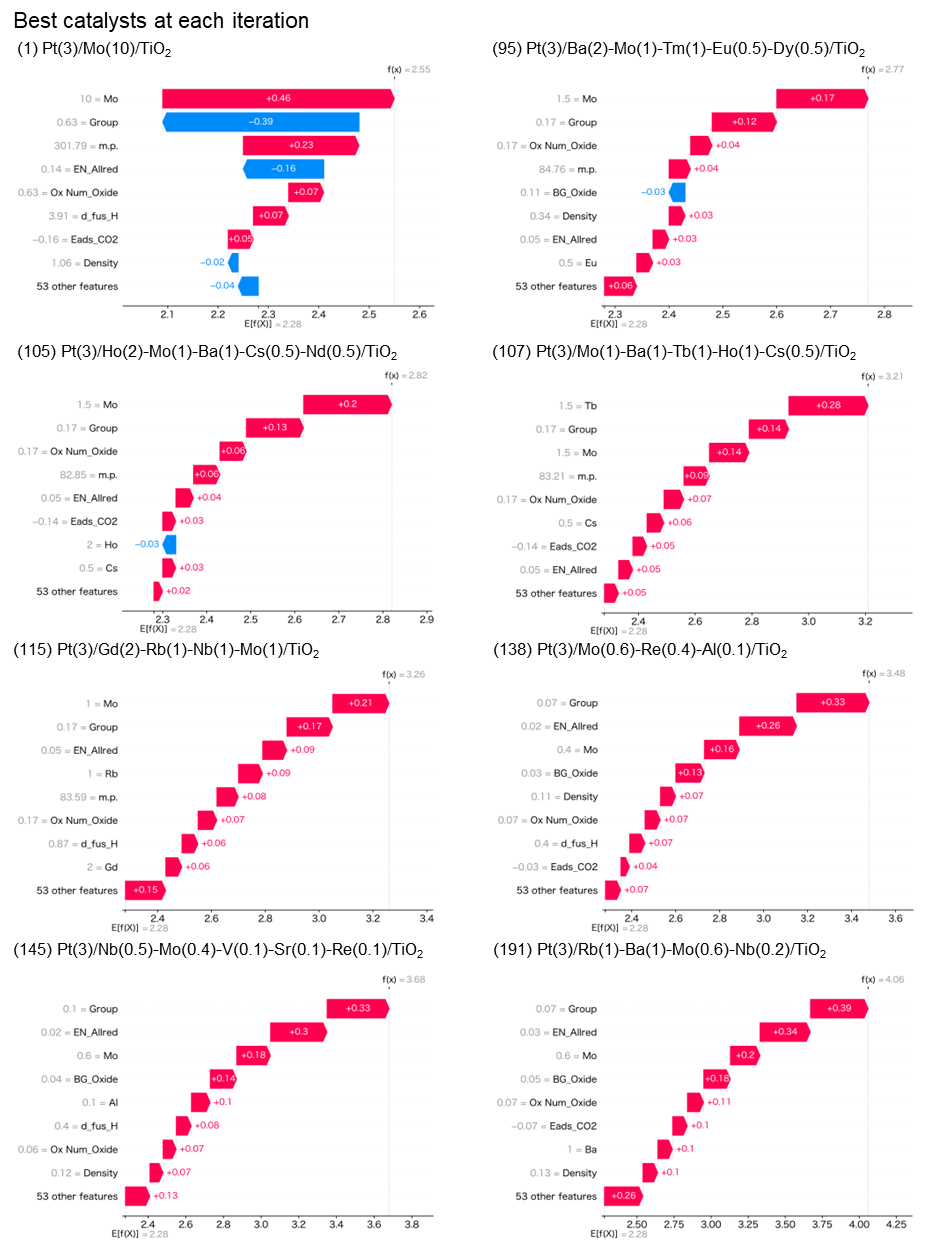
**

**Supplementary Fig. 17.** Breakdown of SHAP values as waterfall plots for the eight catalysts feature in Fig. 1 in the main text to determine which feature values are responsible for increases and decreases from the base value (dataset average: E[f(X)]) of 2.28 relative to the predicted value. Positive and negative contributions of each feature (SHAP values: f(x)) are shown in red and blue, respectively. The exploitative elemental descriptor representation was used.


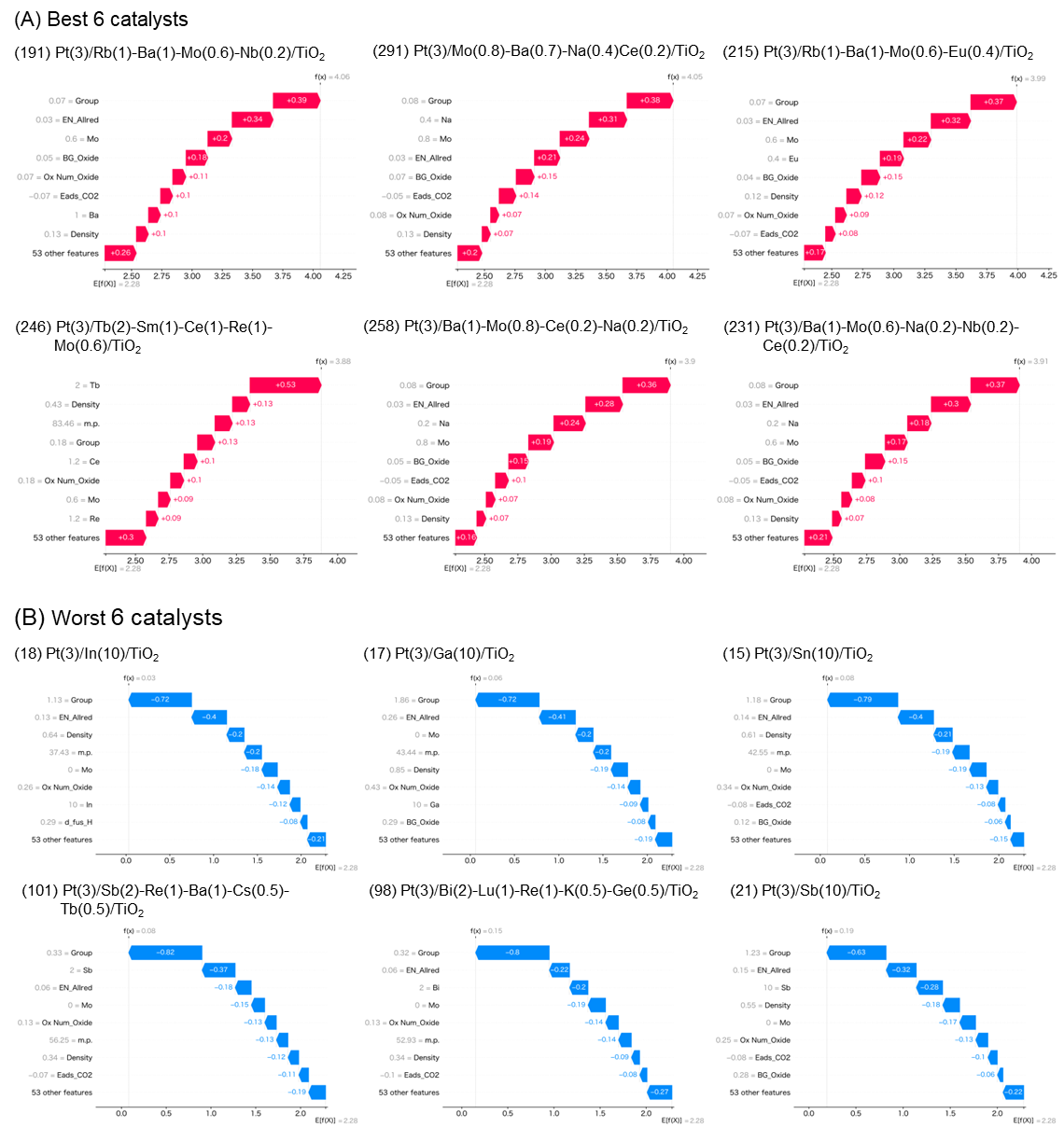


**Supplementary Fig. 18.** Breakdown of SHAP values as waterfall plots for the (A) best six catalysts and (B) worst six catalysts to determine which feature values are responsible for increases and decreases from the base value (dataset average: E[f(X)]) of 2.28 relative to the predicted value. Positive and negative contributions of each feature (SHAP values: f(x)) are shown in red and blue, respectively. The exploitative elemental descriptor representation was used.

**Supplementary Text 4 (Catalyst Characterization)**

The Brunauer–Emmett–Teller (BET) specific surface areas (S_BET_) of these samples were determined by evaluating physical N_2_ adsorption isotherms, and the results are summarized in Supplementary Table 6, with the Pt particle size estimated through low-temperature CO pulse experiments and STEM observations.

Results of XAS and XPS were confirmed by TPR experiments using H_2_ (Supplementary Fig. 25). A peak originating from the reduction of Pt species was observed at approximately -10 °C. Our previous study on a similar material demonstrated that the Pt L_3_-edge XANES of Pt(3)/Mo(20)/TiO_2_ after H_2_ reduction at room temperature was identical to that of metallic Pt foil^42^, corroborating this conclusion. A relatively small peak can be noticed at approximately 270 °C in the H_2_-TPR profile. Since the reduced Mo species were identified by XAS and XPS analyses, this peak was assigned to the reduction of Mo oxides. A very broad reduction peak around 400-600 °C and the other one at above 800 °C were also observed, indicating the further reduction of the Mo species at those temperatures. These results suggest that the supported Pt species promote the reduction of Mo oxide species and the introduced additive components favor the good dispersion of Mo species which can also facilitate the reduction of Mo oxides.

In addition to CO adsorption IR spectra (Fig. 4D), XPS spectra of the Pt 4*f* region were also collected on the samples of Pt(3)/Rb(1)-Ba(1)-Mo(0.6)-Nb(0.2)/TiO_2_, Pt(3)/Rb(1)Mo(0.6)Ba(1)/TiO_2_, Pt(3)/Mo(0.6)/TiO_2_ and Pt(3)/TiO_2_ without exposure to air (Supplementary Fig. 27) to confirm the electronic state of the Pt species in the catalysts. The peak centers of the 4*f*7/2 signals are observed at the binding energies of 71.5, 71.4, 71.2 and 70.7 eV, respectively. All of these peaks can be assigned to metallic Pt ^43^ that shifts to higher energy in the order of Pt(3)/TiO_2_, Pt(3)/Rb(1)Mo(0.6)Ba(1)/TiO_2_, Pt(3)/Mo(0.6)/TiO_2_, and Pt(3)/Rb(1)-Ba(1)-Mo(0.6)-Nb(0.2)/TiO_2_. These observed results are consistent with the results obtained from IR measurements for CO adsorption. Although a negative shift is observed for Pt(3)/TiO_2_ compared to the Pt 4*f*7/2 binding energy of bulk metallic Pt^0^ (71.2 eV) ^43^ that was reported previously for similar Pt/TiO_2_ materials, ^44–46^ the introduction of additive component species causes this peak to shift to a higher binding energy owing to the electron transfer from the support to Pt. ^44,45^


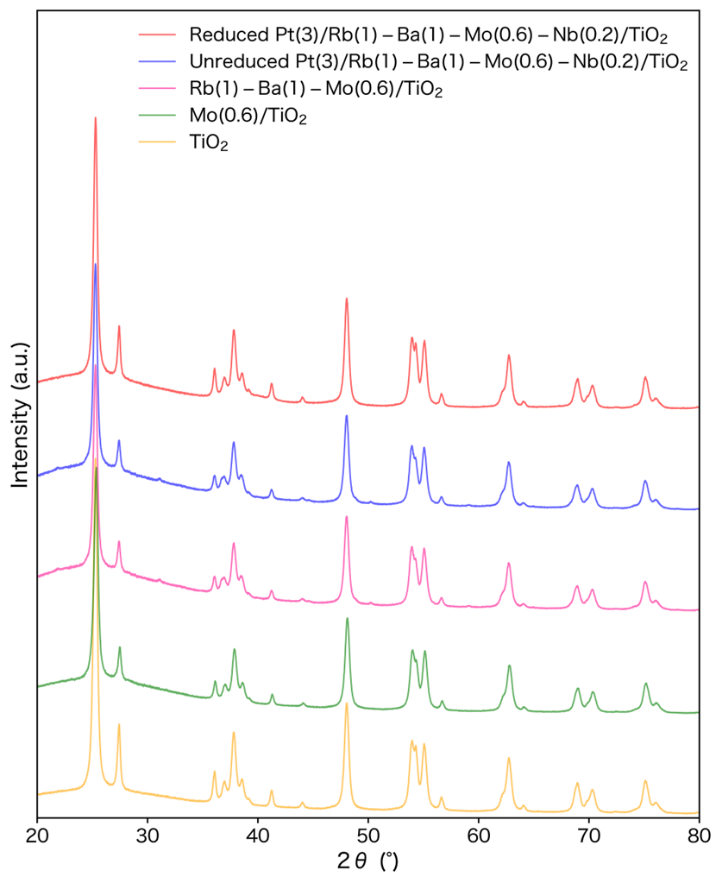


**Supplementary Fig. 19.** XRD patterns of pristine TiO_2_, Mo(0.6)/TiO_2_, Rb(1)-Ba(1)-Mo(0.6)-Nb(0.2)/TiO_2_, unreduced Pt(3)/Rb(1)-Ba(1)-Mo(0.6)-Nb(0.2)/TiO_2_, and Pt(3)/Rb(1)-Ba(1)-Mo(0.6)-Nb(0.2)/TiO_2_.

**Supplementary Table 6.** Textural properties of Rb(1)-Ba(1)-Mo(0.6)-Nb(0.2)/TiO_2_ support, unreduced Pt(3)/Rb(1)-Ba(1)-Mo(0.6)-Nb(0.2)/TiO_2_, and reduced Pt(3)/Rb(1)-Ba(1)-Mo(0.6)-Nb(0.2)/TiO_2_.

| Catalyst | Specific surface area /(m^2^ g^-1^) *^a^* | Pt particle size /nm *^b^* | Pt particle size /nm *^c^* |
| --- | --- | --- | --- |
| Rb(1)-Ba(1)-Mo(0.6)-Nb(0.2)/TiO_2_ | 50.7 | - | - |
| Unreduced Pt(3)/Rb(1)-Ba(1)-Mo(0.6)-Nb(0.2)/TiO_2_ | 50.2 | - | - |
| Pt(3)/Rb(1)-Ba(1)-Mo(0.6)-Nb(0.2)/TiO_2_ | 50.7 | 2.9 | 1.8 |

*^a^* Determined by N_2_ adsorption, *^b^* estimated by CO adsorption at −20 °C, *^c^* estimated by STEM.


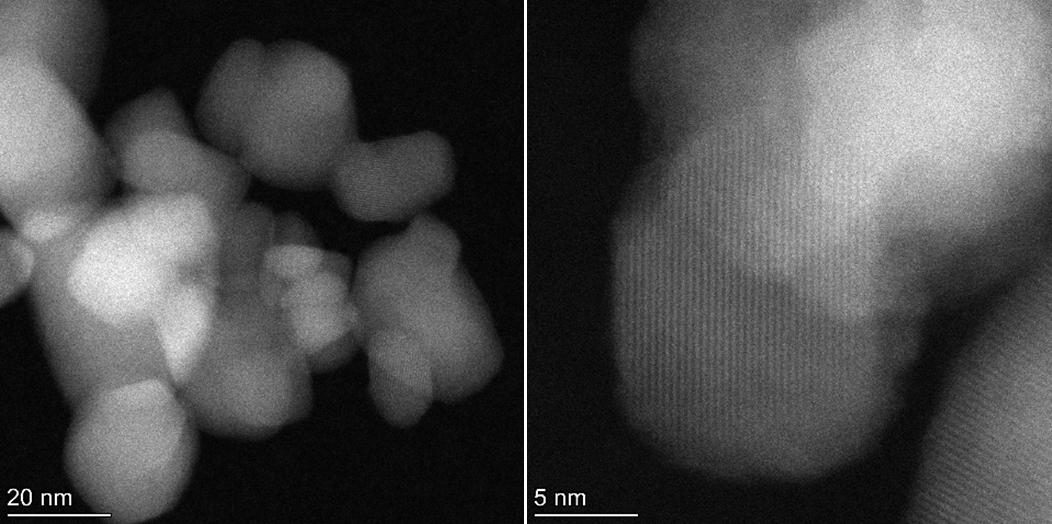


**Supplementary Fig. 20.** HAADF-STEM images of TiO_2_.


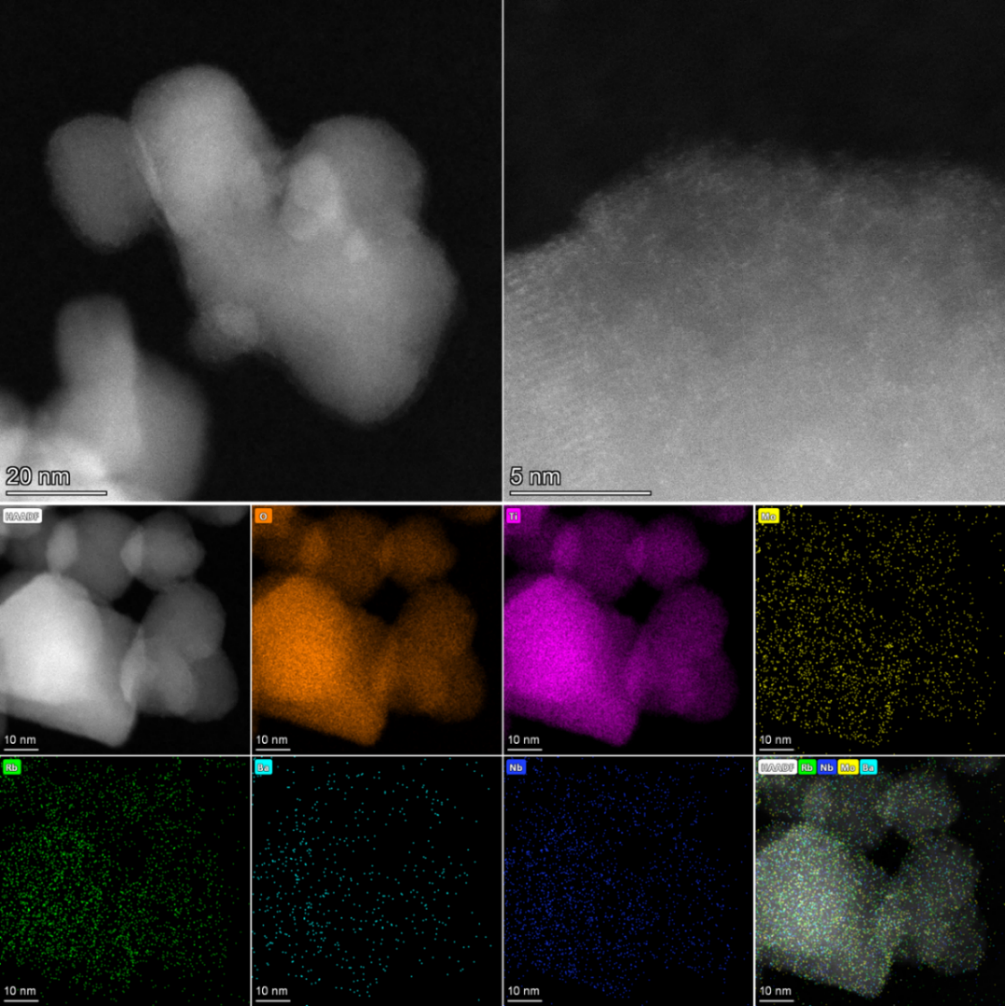


**Supplementary Fig. 21.** HAADF-STEM images and EDX mapping of the Rb(1)-Ba(1)-Mo(0.6)-Nb(0.2)/TiO_2_ support.


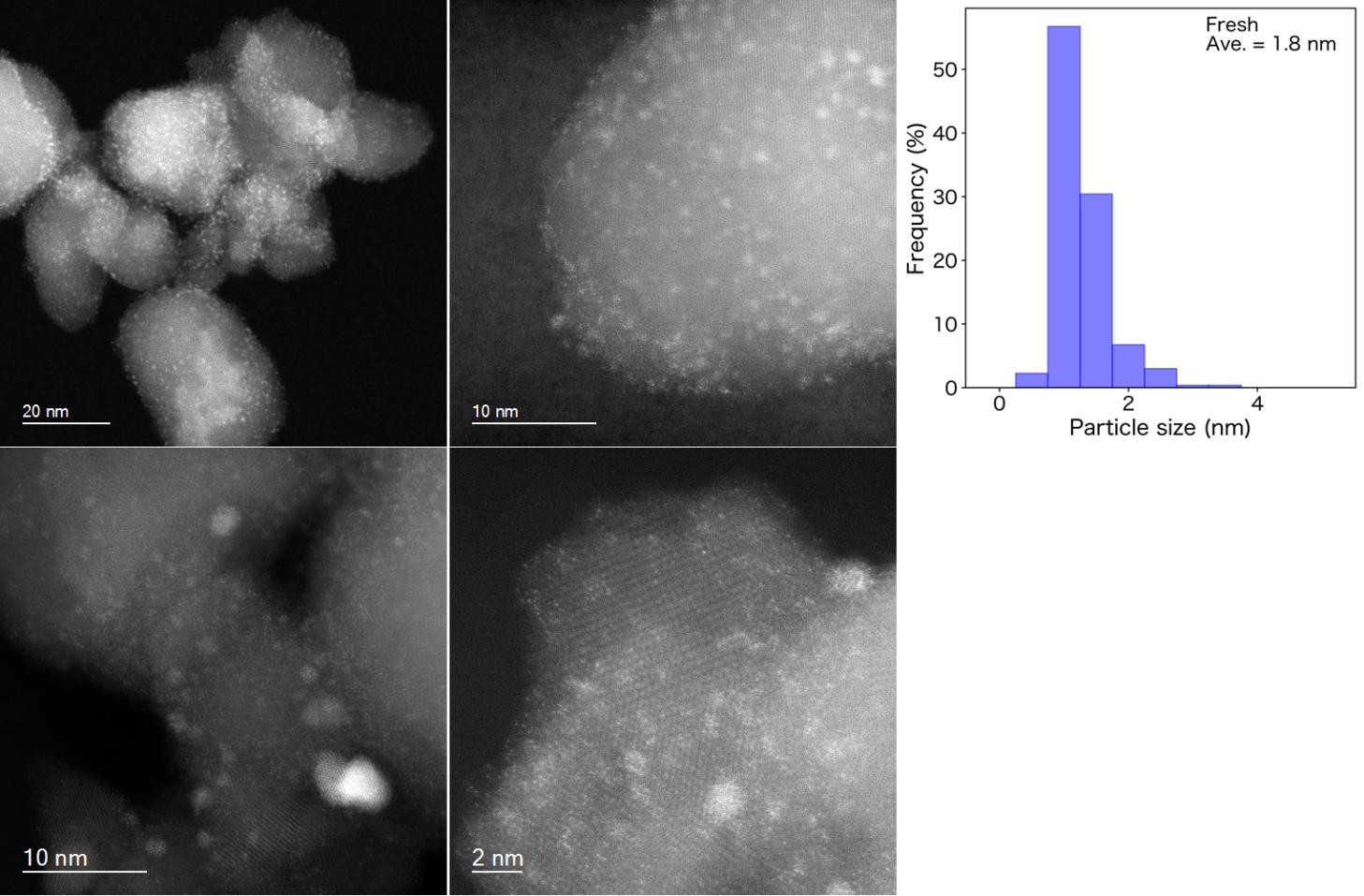


**Supplementary Fig. 22.** HAADF-STEM images and Pt particle size distribution of the optimal Pt(3)/Rb(1)-Ba(1)-Mo(0.6)-Nb(0.2)/TiO_2_ catalyst.


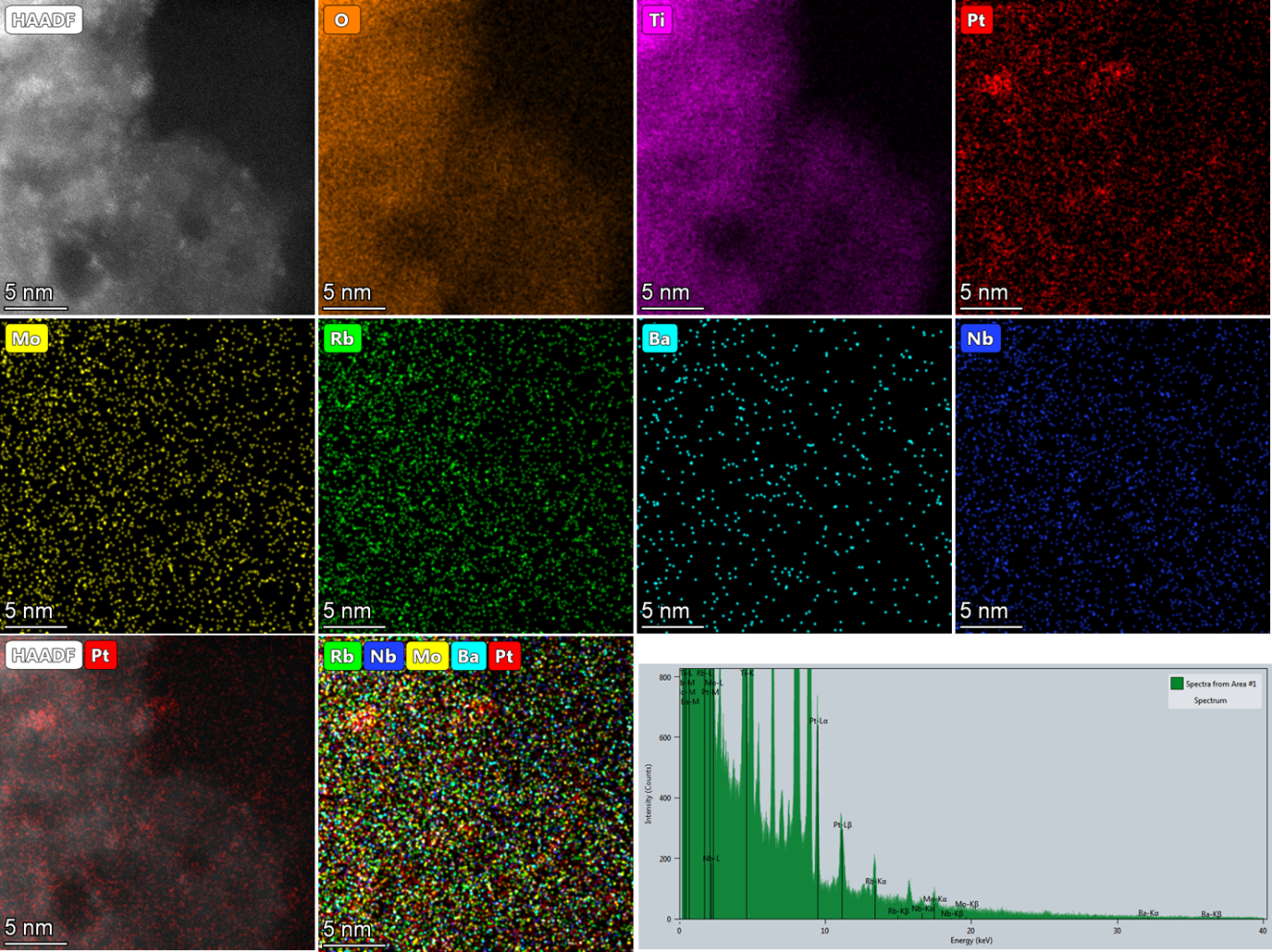


**Supplementary Fig. 23.** HAADF-STEM image and EDX mapping of the optimal Pt(3)/Rb(1)-Ba(1)-Mo(0.6)-Nb(0.2)/TiO_2_ catalyst.

**Supplementary Table 7.** Pt L_3_-edge EXAFS curve fitting analysis for unreduced and reduced Pt(3)/Rb(1)-Ba(1)-Mo(0.6)-Nb(0.2)/TiO_2_.

| Catalyst | Shell | CN ^a^ | *R* (Å) ^b^ | σ^2^ (Å^2^) ^c^ | R_f_ (%) ^d^ |
| --- | --- | --- | --- | --- | --- |
| Unreduced Pt(3)/Rb(1)-Ba(1)-Mo(0.6)-Nb(0.2)/TiO_2_ | Pt–O | 5.6 | 2.01 | 0.004 | 1.3 |
| Pt(3)/Rb(1)-Ba(1)-Mo(0.6)-Nb(0.2)/TiO_2_ | Pt–Pt | 5.6 | 2.75 | 0.008 | 1.1 |

^a^ Coordination number. ^b^ Bond distance. ^c^ Debye-Waller factor. ^d^ Residual factor.


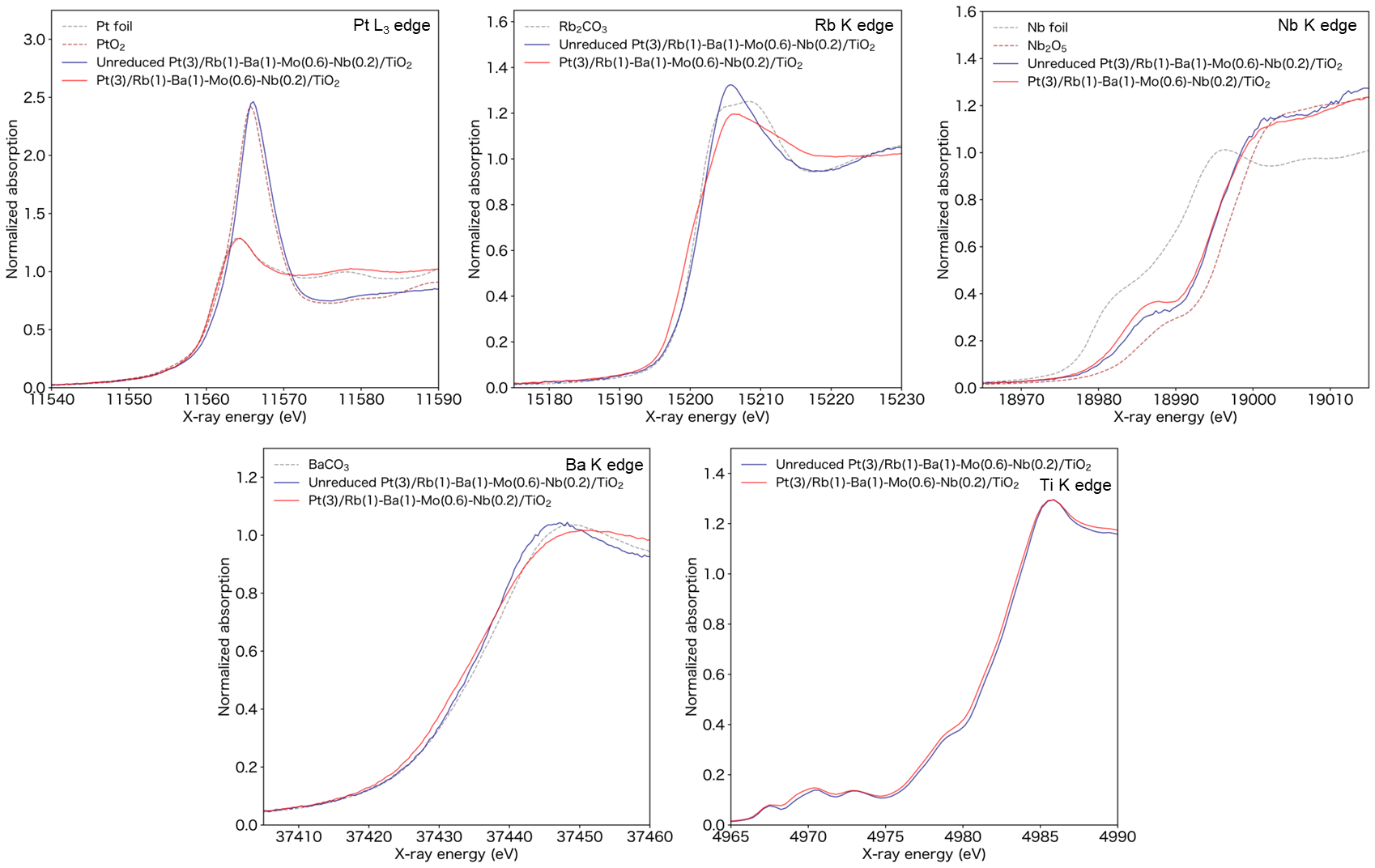


**Supplementary Fig. 24.** XANES of unreduced Pt(3)/Rb(1)-Ba(1)-Mo(0.6)-Nb(0.2)/TiO_2_, Pt(3)/Rb(1)-Ba(1)-Mo(0.6)-Nb(0.2)/TiO_2_ (reduced), and reference compounds. The spectrum of Pt(3)/Rb(1)-Ba(1)-Mo(0.6)-Nb(0.2)/TiO_2_ (reduced) was collected *in situ* at 250 °C just after H_2_ reduction at 300 °C, and the others were recorded at room temperature.


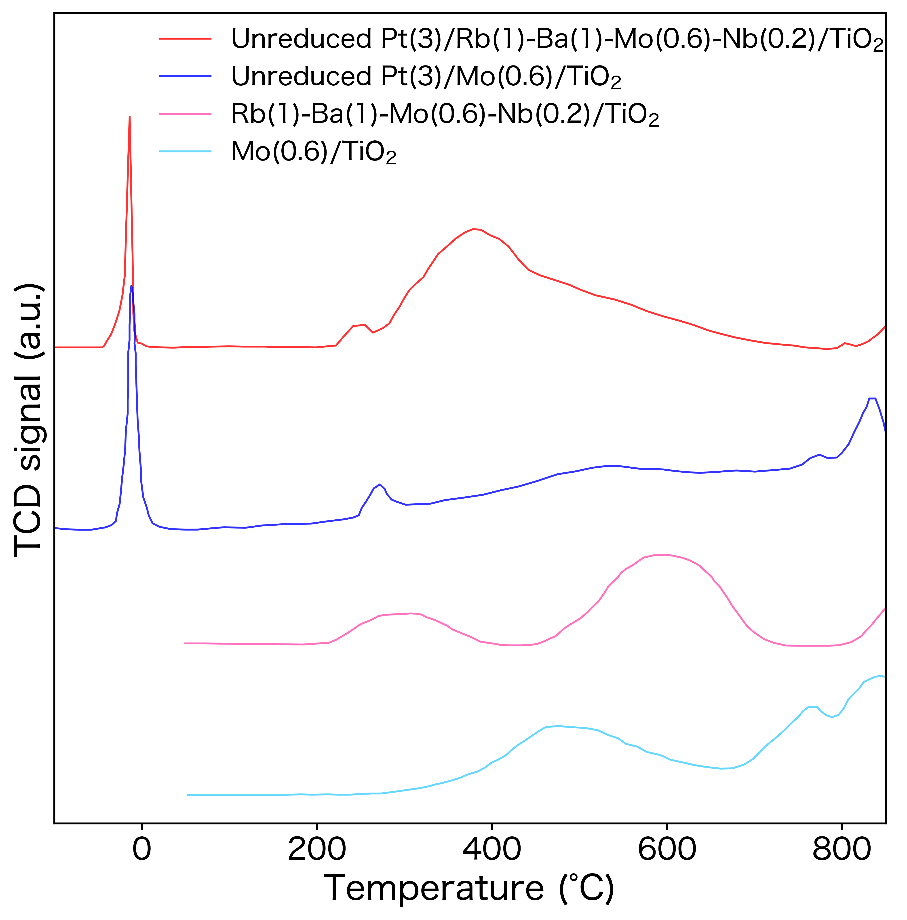


**Supplementary Fig. 25.** H_2_-TPR profiles of various unreduced Mo(0.6)/TiO_2_, Rb(1)-Ba(1)-Mo(0.6)-Nb(0.2)/TiO_2_, Pt(3)/Mo(0.6)/TiO_2_ and Pt(3)/Rb(1)-Ba(1)-Mo(0.6)-Nb(0.2)/TiO_2_. Each sample was heated at a temperature ramp rate of 10 °C min^-1^ under a flow of 5% H_2_/Ar (20 mL min^-1^).


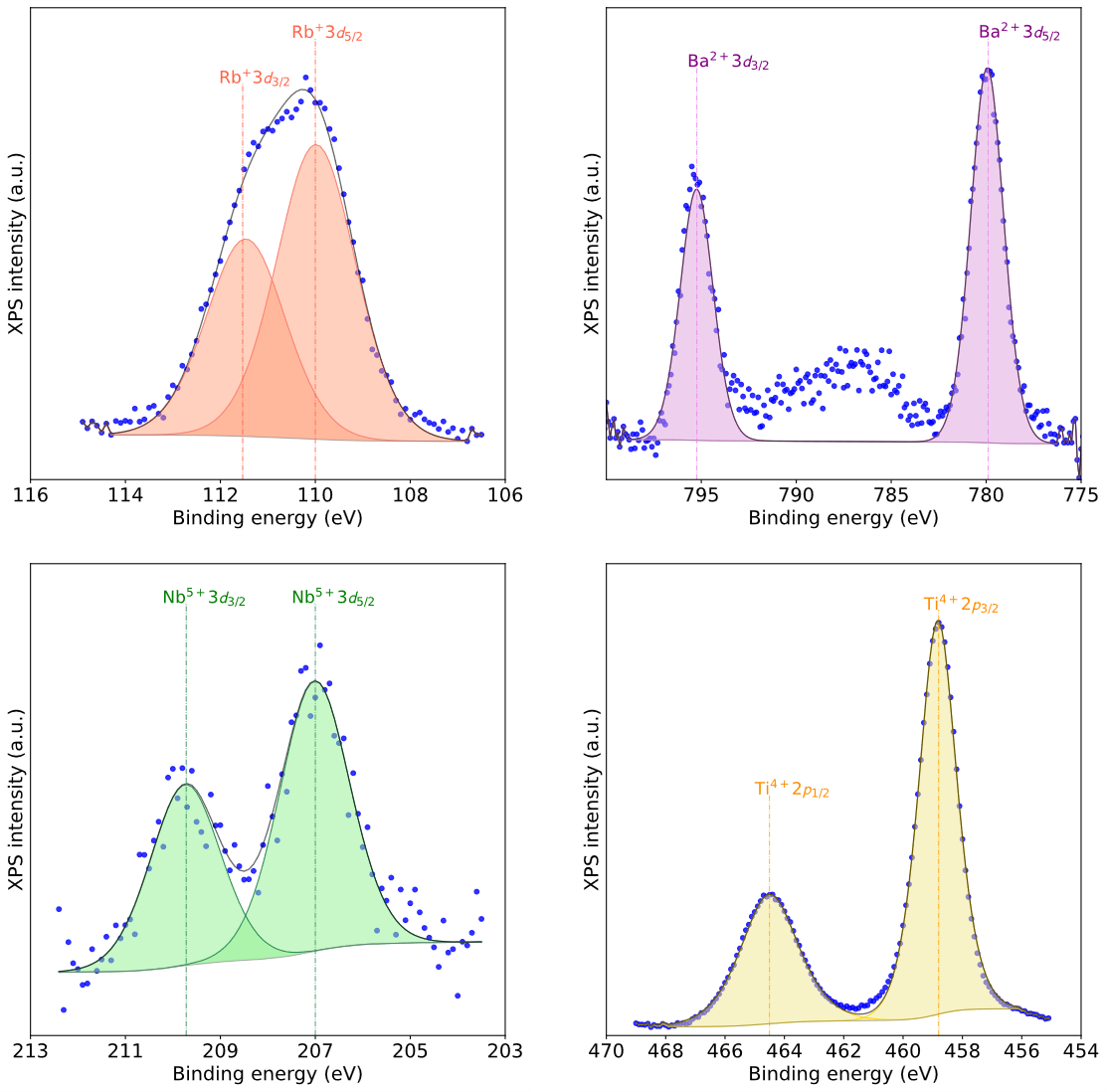


**Supplementary Fig. 26.** XPS spectra of the Rb 3*d*, Ba 3*d*, Nb 3*d*, and Ti *2p* regions of reduced Pt(3)/Rb(1)-Ba(1)-Mo(0.6)-Nb(0.2)/TiO_2_. The sample was measured without exposure to air after H_2_ reduction.


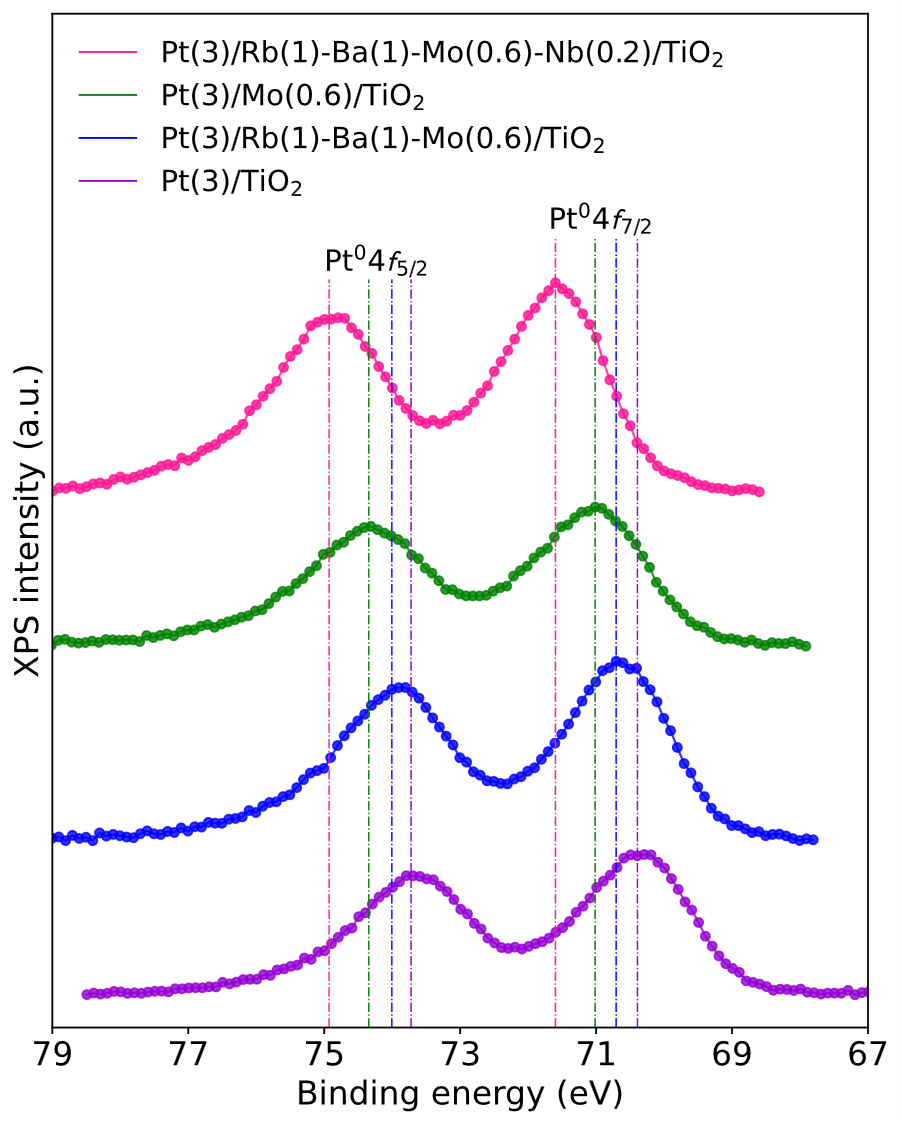


**Supplementary Fig. 27.** XPS spectra of the Pt 4*f* region of supported Pt catalysts after the H_2_ reduction pretreatment at 300 °C without exposure to air.

**Supplementary Text 5 (Mechanistic Studies)**

For comparison to the *operando* IR measurements on Pt(3)/Rb(1)-Ba(1)-Mo(0.6)-Nb(0.2)/TiO_2_, we also measured the Pt(3)/Mo(0.6)/TiO_2_ catalyst to investigate the effect of additive components of Rb, Ba, and Nb that exist in the form of Rb_2_O, BaO and Nb_2_O_5_ in the catalyst during the RWGS reaction process (Supplementary Fig. 33). Evidently, without the beneficial effects of the above-mentioned additive components, fewer surface species (including carbonate and formate species) and less CO are produced at each gas switching step than those on Pt(3)/Rb(1)-Ba(1)-Mo(0.6)-Nb(0.2)/TiO_2_. Therefore, adding Rb, Ba, and Nb can promote not only the direct CO_2_ dissociation but also surface species formation, thus contributing to the production of CO.


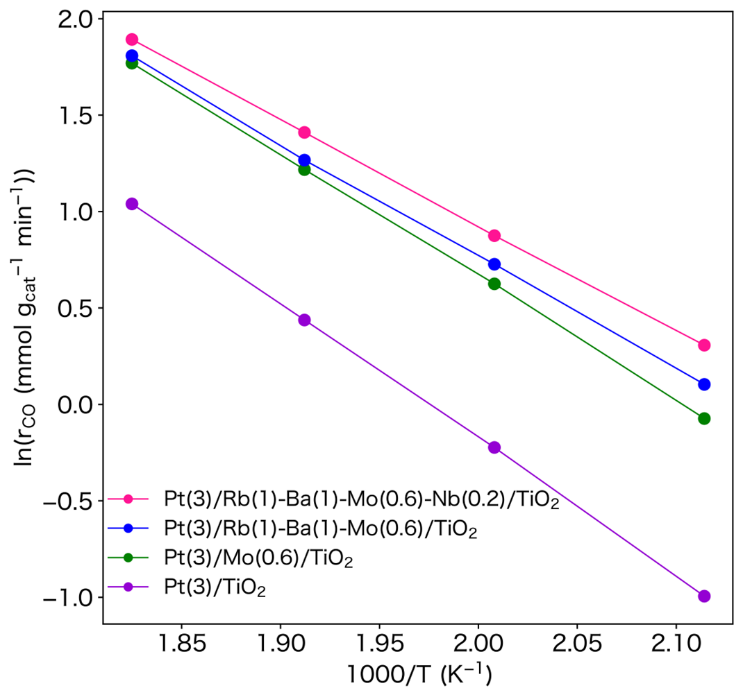


**Supplementary Fig. 28.** Arrhenius plots for the RWGS reaction conducted over Pt(3)/Rb(1)-Ba(1)-Mo(0.6)-Nb(0.2)/TiO_2_, Pt(3)/Rb(1)-Ba(1)-Mo(0.6)/TiO_2_, Pt(3)/Mo(0.6)/TiO_2_, and Pt(3)/TiO_2_ under the following reaction conditions: 10 mg of catalyst, 20 mL min^-1^ CO_2_, 60 mL min^-1^ H_2_, 5 mL min^-1^ N_2_, and 200–275 °C.


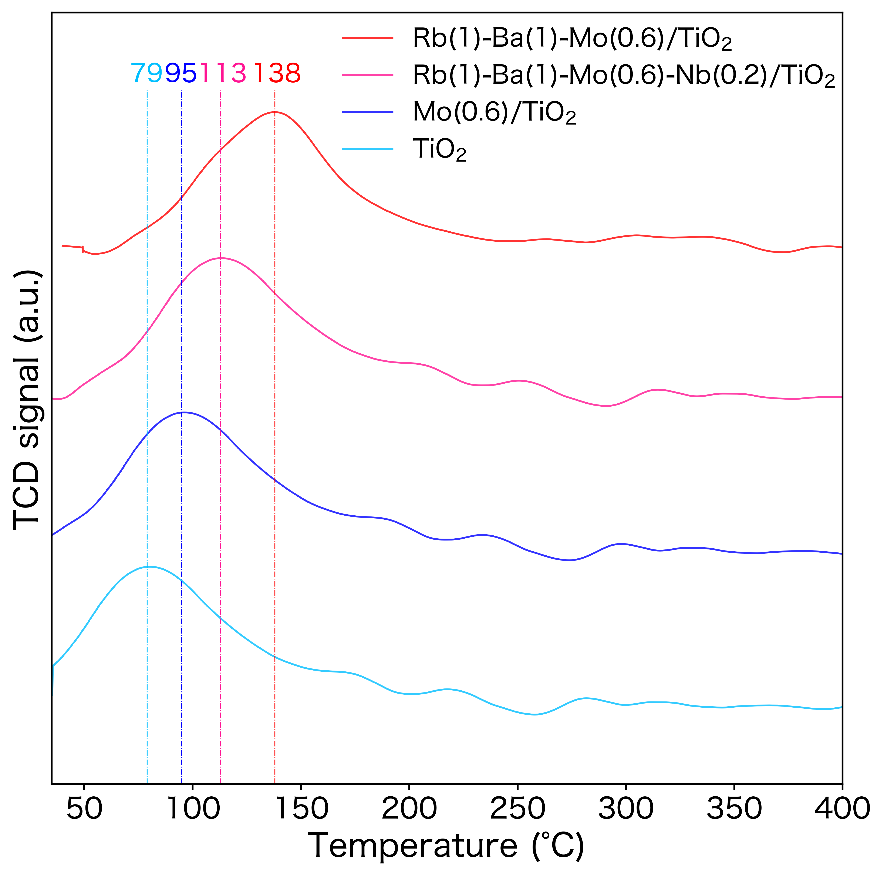


**Supplementary Fig. 29.** CO_2_-TPD of pristine and additive-modified TiO_2_.


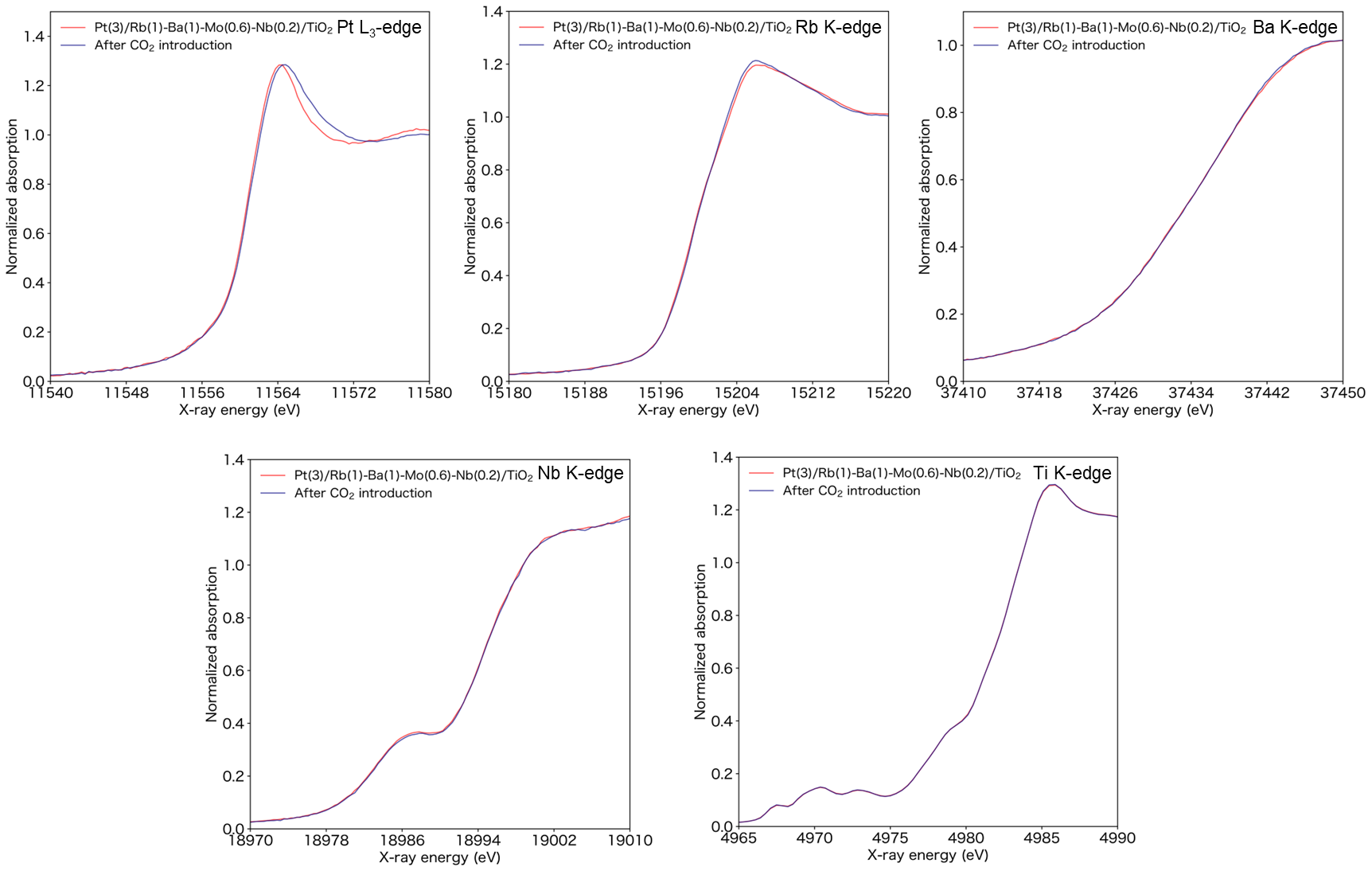


**Supplementary Fig. 30.** *In situ* Pt L_3_-edge, Ti K-edge, Ba K-edge, Rb K-edge and Nb K-edge XANES of Pt(3)/Rb(1)-Ba(1)-Mo(0.6)-Nb(0.2)/TiO_2_ measured under a sequential flow of 25% CO_2_/He, 75% H_2_/He, and 25% CO_2_ + 75% H_2_ at 250 °C following the H_2_ reduction pretreatment at 300 °C.


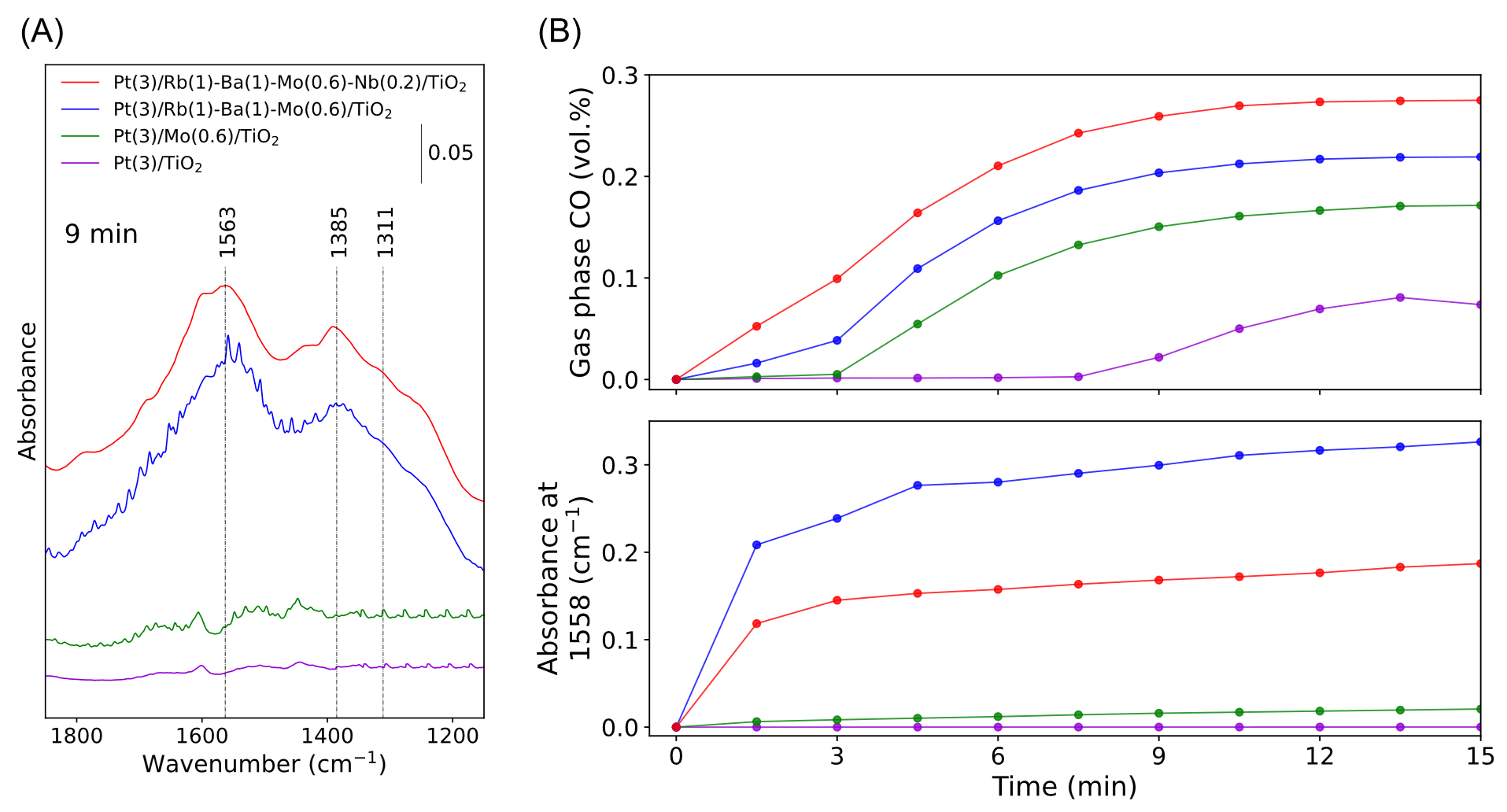


**Supplementary Fig. 31.** (A) *Operando* IR spectra obtained under a flow of 100% CO_2_ at 250 °C after the H_2_ reduction pretreatment at 300 °C and (B) variations in the intensities of peaks related to surface adsorbed species and the concentration of CO in the effluent gas upon the introduction of CO_2_.


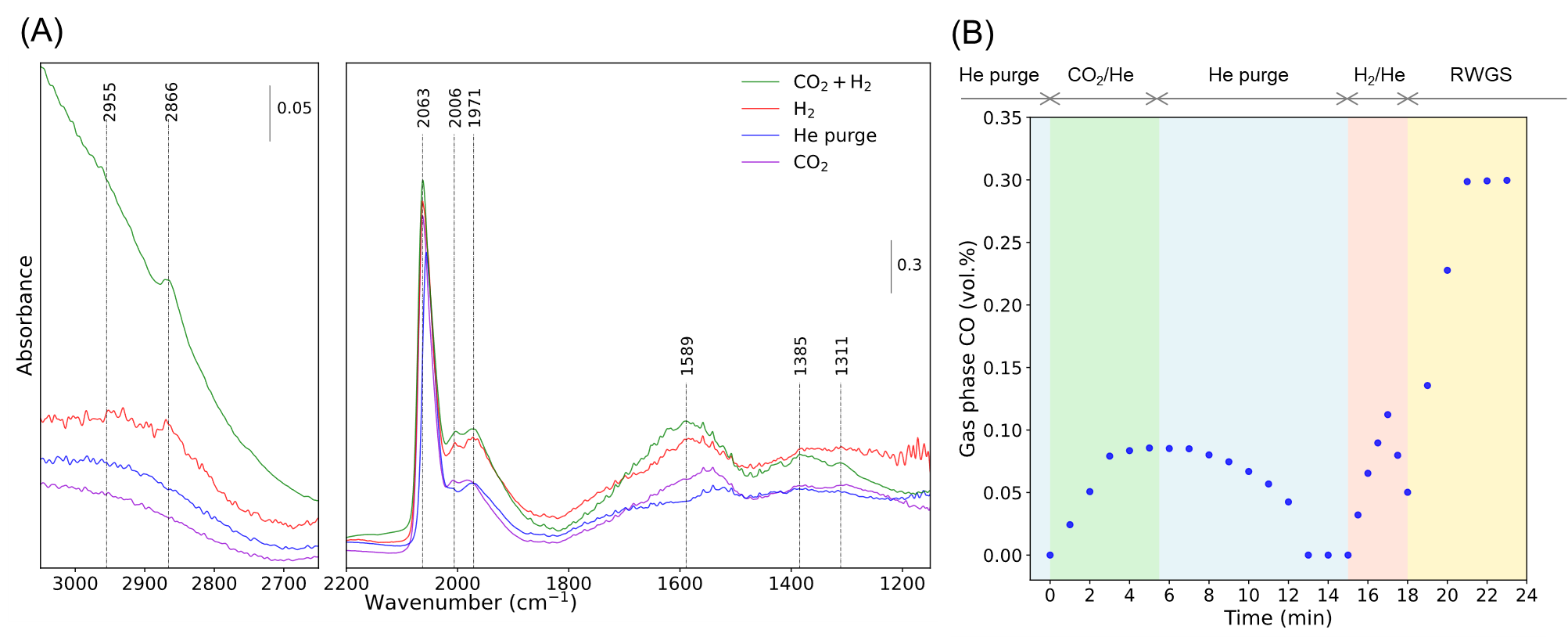


**Supplementary Fig. 32.** (A) *Operando* IR spectra for Pt(3)/Rb(1)-Ba(1)-Mo(0.6)-Nb(0.2)/TiO_2_ catalyst recorded under a sequential flow of CO_2_ (20 mL min^-1^), He (50 mL min^-1^), H_2_ (60 mL min^-1^), and CO_2_ (20 mL min^-1^) + H_2_ (60 mL min^-1^) mixture at 250 °C following the H_2_ reduction pretreatment at 300 °C and (B) Variations in the intensities of peaks related to adsorbed surface species and the concentration of CO in the effluent gas upon the introduction of CO_2_. Owing to the background shift, only the concentration of the gas phase CO is plotted.


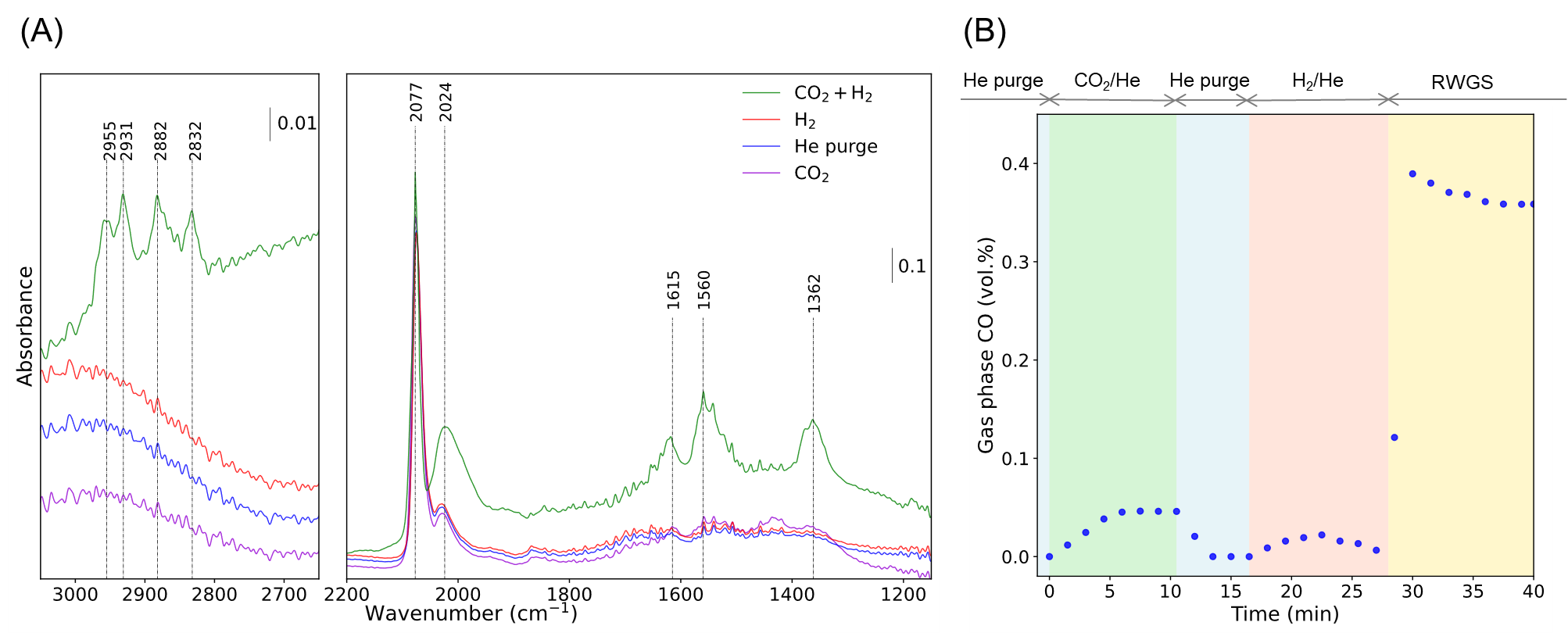


**Supplementary Fig. 33.** (A) *Operando* IR spectra for Pt(3)/Mo(0.6)/TiO_2_ catalyst recorded under a sequential flow of CO_2_ (20 mL min^-1^), He (50 mL min^-1^), H_2_ (60 mL min^-1^), and CO_2_ (20 mL min^-1^) + H_2_ (60 mL min^-1^) mixture at 200 ^o^C following the H_2_ reduction pretreatment at 300 °C and (B) variations in the intensities of peaks related to adsorbed surface species and the concentration of CO in the effluent gas upon the introduction of CO_2_. Owing to the background shift, only the concentration of the gas phase CO is plotted.

**Supplementary Text 6 (Catalyst Stability)**

To elucidate the origin of catalytic deactivation, XRD, HAADF-STEM, and XAS analyses were performed for the Pt(3)/Rb(1)-Ba(1)-Mo(0.6)-Nb(0.2)/TiO_2_ catalyst after 300 h of time-on-stream (Supplementary Fig.s 34–36 and Supplementary Table 8). No significant changes were found in the XRD patterns, and only peaks from TiO_2_ were observed, indicating the absence of the crystalline species that are detectable by XRD besides the TiO_2_ support. The average particle size of the Pt species in the spent catalyst was larger (6.0 nm) than that in the fresh sample (1.8 nm), indicating the aggregation of supported Pt after long-term RWGS reaction. Although no significant changes were observed in the Pt L_3_-edge, Ba K-edge, Rb K-edge, Nb K-edge, or Ti K-edge XANES, the Mo K-edge XANES of the spent catalyst shifted slightly toward higher energies, suggesting the partial oxidation of the Mo species after long-term reaction. In addition, a curve-fitting analysis of the Pt L_3_-edge EXAFS demonstrated that the coordination number of the Pt–Pt bond increased from 5.6 to 6.9 (Supplementary Table 8), indicating that Pt particles aggregated during the RWGS reaction, which is consistent with the HAADF-STEM observations. It should also be noted that the formation of Pt–Mo alloys can also be a possible reason for the deactivation^47,48^, although there is no obvious evidence for this deactivation pathway. Therefore, it can be concluded that the catalytic deactivation of Pt(3)/Rb(1)-Ba(1)-Mo(0.6)-Nb(0.2)/TiO_2_ is caused by the aggregation of Pt particles and a slight change in the oxidation state of Mo.


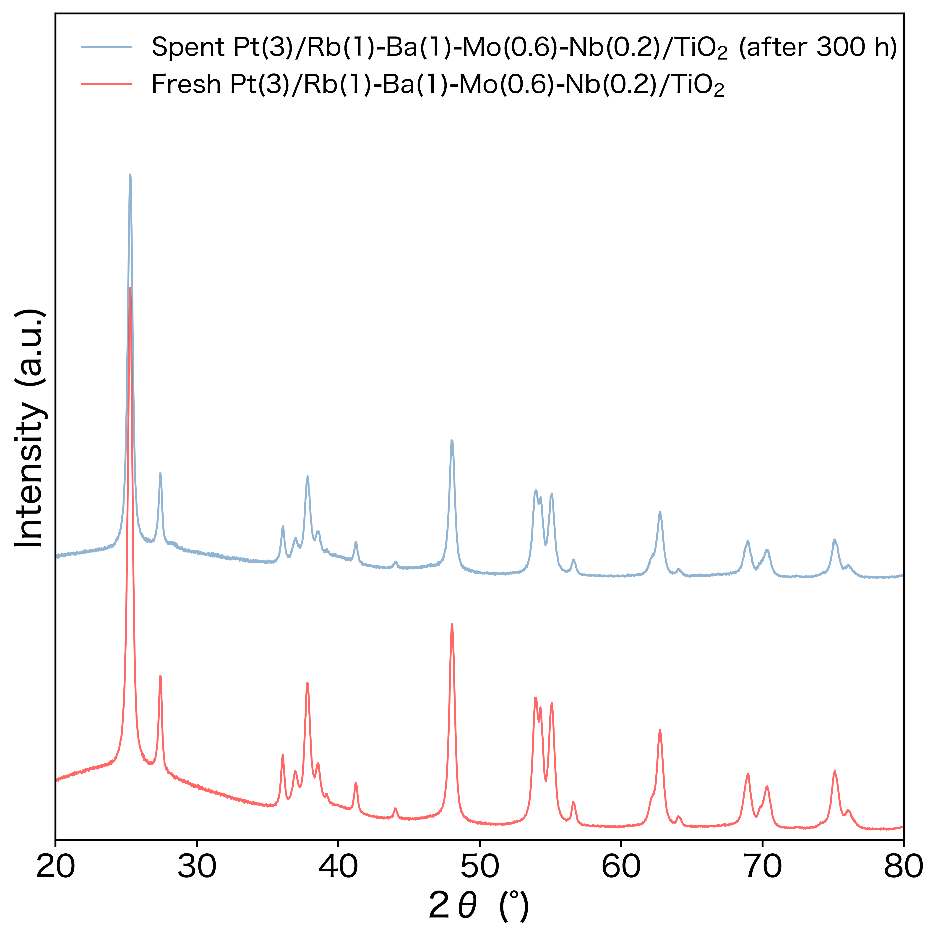


**Supplementary Fig. 34.** XRD patterns of fresh and spent Pt(3)/Rb(1)-Ba(1)-Mo(0.6)-Nb(0.2)/TiO_2_ catalyst.


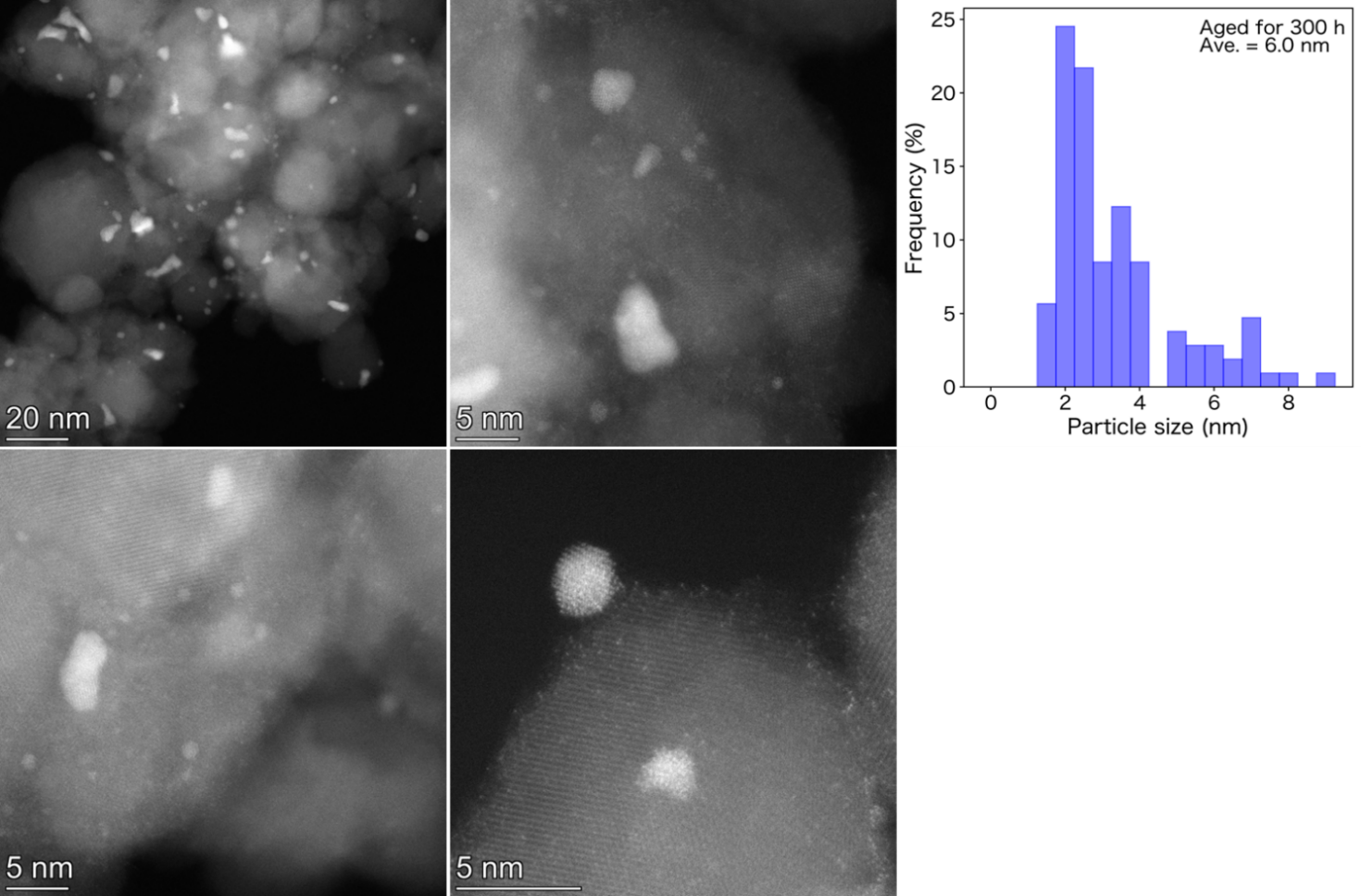


**Supplementary Fig. 35.** HAADF-STEM images and Pt particle size distribution of the spent Pt(3)/Rb(1)-Ba(1)-Mo(0.6)-Nb(0.2)/TiO_2_ catalyst after 300 h time-on-stream.


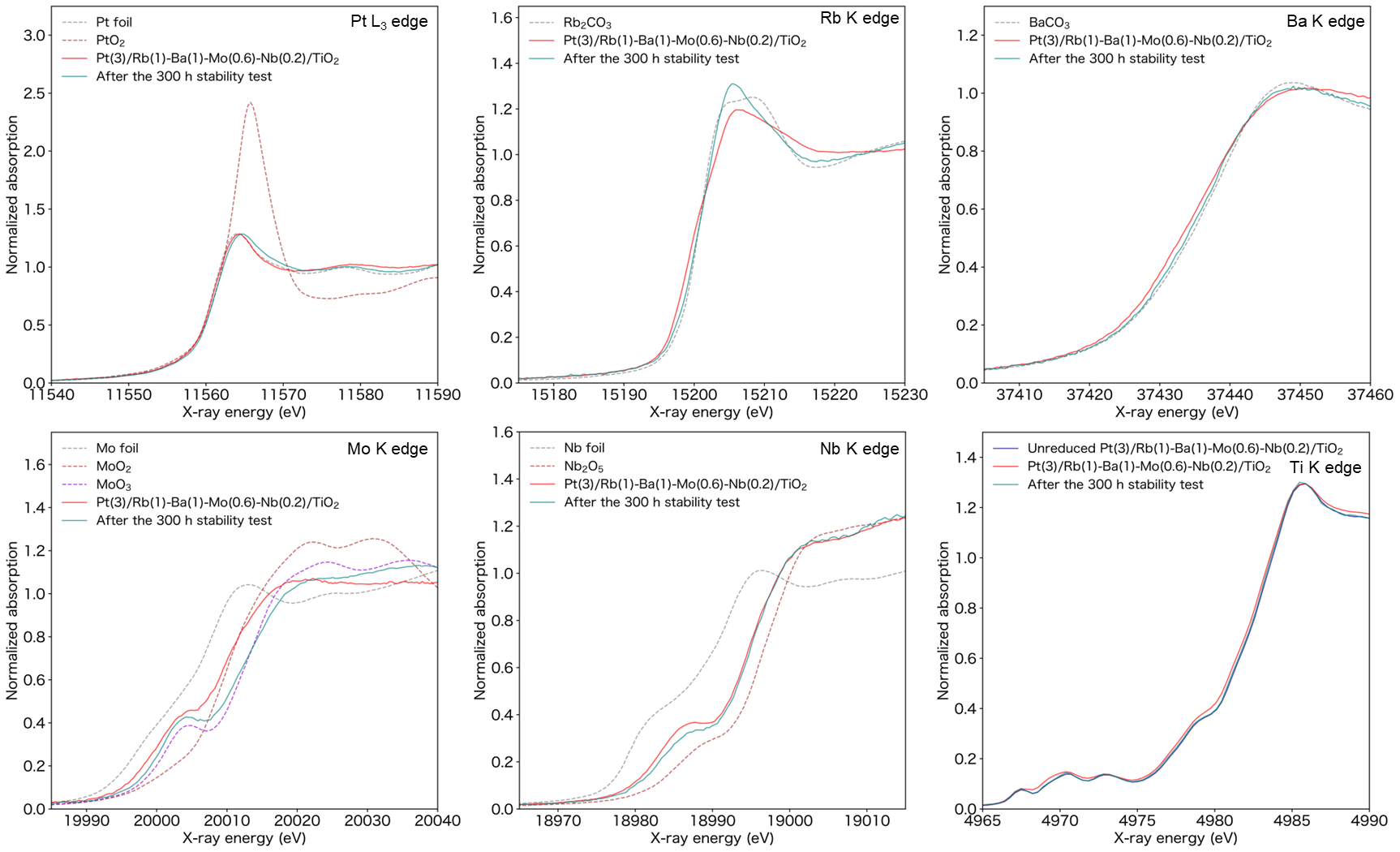


**Supplementary Fig. 36.** Pt L_3_-edge, Mo K-edge, Rb K-edge, Ba K-edge, Nb K-edge, and Ti K-edge XANES spectra of fresh and spent Pt(3)/Rb(1)-Ba(1)-Mo(0.6)-Nb(0.2)/TiO_2_ catalyst and related reference samples. The XAS spectra were recorded at room temperature without exposure to air after the reduction/reaction treatment.

**Supplementary Table 8.** Pt L_3_-edge EXAFS curve fitting analysis for the Pt(3)/Rb(1)-Ba(1)-Mo(0.6)-Nb(0.2)/TiO_2_ catalyst before (fresh) and after 300 h reaction (spent).

| Catalyst | Shell | CN ^a^ | *R* (Å) ^b^ | σ^2^ (Å^2^) ^c^ | R_f_ (%) ^d^ |
| --- | --- | --- | --- | --- | --- |
| Fresh | Pt–Pt | 5.6 | 2.75 | 0.008 | 1.1 |
| Spent | Pt–Pt | 6.9 | 2.75 | 0.006 | 0.2 |

^a^ Coordination number. ^b^ Bond distance. ^c^ Debye-Waller factor. ^d^ Residual factor.

**Supplementary References**

1. Kresse, G. & Furthmüller, J. Efficient iterative schemes for ab initio total-energy calculations using a plane-wave basis set. *Phys. Rev. B - Condens. Matter Mater. Phys.* **54**, 11169–11186 (1996).

2. Kresse, G. & Furthmüller, J. Efficiency of ab-initio total energy calculations for metals and semiconductors using a plane-wave basis set. *Comput. Mater. Sci.* **6**, 15–50 (1996).

3. Perdew, J. P. *et al.* Restoring the density-gradient expansion for exchange in solids and surfaces. *Phys. Rev. Lett.* **100**, 136406 (2008).

4. Blöchl, P. E. Projector augmented-wave method. *Phys. Rev. B* **50**, 17953–17979 (1994).

5. Grimme, S., Ehrlich, S. & Goerigk, L. Effect of the damping function in dispersion corrected density functional theory. *J. Comput. Chem.* **32**, 1456–1465 (2011).

6. Shrinkage, R. Regression Shrinkage and Selection via the Lasso. *J. R. Stat. Soc. B* **58**, 267–288 (1996).

7. Hoerl, A. E. & Kennard, R. W. Ridge Regression: Applications to Nonorthogonal Problems. *Technometrics* **12**, 69–82 (1970).

8. R. H. Myers, D. C. Montgomery, C. M. A.-C. *Response Surface Methodology: Process and Product Optimization Using Designed Experiments*. **001**, (1985).

9. Drucker, H., Burges, C. J. C. C., Kaufman, L., Smola, A. & Vapnik, V. Support vector regression machines. *Adv. Neural Inf. Process. Syst.* **9**, 155–161 (1997).

10. Breiman, L. Random forests. *Mach. Learn.* **45**, 5–32 (2001).

11. Pedregosa, F. & Varoquaux, G. Scikit-learn: Machine learning in Python. *J. Mach. Learn. Res* **12**, 2825–2830 (2011).

12. Allred, A. L. & Rochow, E. G. A Scale of Electronegativity Based on Electrostatic Force. *J. Inorg. Nucl. Chem* **5**, 264–268 (1958).

13. Lide, D. R. CRC Handbook of Chemistry and Physics, 84th Edition, 2003-2004. *Handb. Chem. Phys.* **53**, 2616 (2003).

14. Goguet, A., Meunier, F. C., Tibiletti, D., Breen, J. P. & Burch, R. Spectrokinetic investigation of reverse water-gas-shift reaction intermediates over a Pt/CeO_2_ catalyst. *J. Phys. Chem. B* **108**, 20240–20246 (2004).

15. Xu, Y. F. *et al.* High-performance light-driven heterogeneous CO_2_ catalysis with near-unity selectivity on metal phosphides. *Nat. Commun.* **11**, 5149 (2020).

16. Hu, X., Hu, X., Guan, Q. & Li, W. Adjusting the active sites of Cu and ZnO by coordination effect of H_3_BTC and its influence on enhanced RWGS reaction. *Sustain. Energy Fuels* **4**, 2937–2949 (2020).

17. Kitamura, K., Soga, K., Kunimori, K. & Arakawa, H. Effect of Li additive on CO_2_ hydrogenation reactivity of zeolite supported Rh catalysts. **175**, 67–81 (1998).

18. Wang, C. *et al.* Product Selectivity Controlled by Nanoporous Environments in Zeolite Crystals Enveloping Rhodium Nanoparticle Catalysts for CO_2_ Hydrogenation. *J. Am. Chem. Soc.* **141**, 8482−8488 (2019).

19. Yang, X. *et al.* Promotion effects of potassium on the activity and selectivity of Pt/zeolite catalysts for reverse water gas shift reaction. *Appl. Catal. B Environ.* **216**, 95–105 (2017).

20. Gonçalves, R. V. *et al.* Selective hydrogenation of CO_2_ into CO on a highly dispersed nickel catalyst obtained by magnetron sputtering deposition: A step towards liquid fuels. *Appl. Catal. B Environ.* **209**, 240–246 (2017).

21. Chen, Y., Hong, H., Cai, J. & Li, Z. Highly Efficient CO_2_ to CO Transformation over Cu‐Based Catalyst Derived from a CuMgAl‐Layered Double Hydroxide (LDH). *ChemCatChem* 13, 656–663 (2020).

22. Bobadilla, L. F., Santos, J. L., Ivanova, S., Odriozola, J. A. & Urakawa, A. Unravelling the Role of Oxygen Vacancies in the Mechanism of the Reverse Water-Gas Shift Reaction by Operando DRIFTS and Ultraviolet-Visible Spectroscopy. *ACS Catalysis* **8**, 7455−7467 (2018).

23. Chen, X. *et al.* Catalytic performance of the Pt/TiO_2_ catalysts in reverse water gas shift reaction: Controlled product selectivity and a mechanism study. *Catal. Today* **281**, 312–318 (2017).

24. Zhang, J., Deo, S., Janik, M. J. & Will Medlin, J. Control of Molecular Bonding Strength on Metal Catalysts with Organic Monolayers for CO_2_ Reduction. *J. Am. Chem. Soc.* **142**, 5184–5193 (2020).

25. Chen, X. *et al.* Identification of relevant active sites and a mechanism study for reverse water gas shift reaction over Pt/CeO_2_ catalysts. *J. Energy Chem.* **25**, 1051–1057 (2016).

26. Wang, W. *et al.* Reverse water gas shift over In_2_O_3_-CeO_2_ catalysts. *Catal. Today* **259**, 402–408 (2016).

27. Ye, J., Ge, Q. & Liu, C. J. Effect of PdIn bimetallic particle formation on CO reduction over the Pd-In/SiO_2_ catalyst. *Chem. Eng. Sci.* **135**, 193–201 (2015).

28. Tang, R. *et al.* Ru-Catalyzed Reverse Water Gas Shift Reaction with Near-Unity Selectivity and Superior Stability. *ACS Mater. Lett.* **3**, 1652–1659 (2021).

29. Wang, K. *et al.* Operando DRIFTS-MS investigation on plasmon-thermal coupling mechanism of CO_2_ hydrogenation on Au/TiO_2_: The enhanced generation of oxygen vacancies. *Appl. Catal. B Environ.* **296**, 120341 (2021).

30. Wang, Y., Arandiyan, H., Scott, J., Aguey-Zinsou, K. F. & Amal, R. Single Atom and Nanoclustered Pt Catalysts for Selective CO_2_ Reduction. *ACS Appl. Energy Mater.* **1**, 6781–6789 (2018).

31. Kumar, A., Mohammed, A. A. A., Saad, M. A. H. S. & Al-Marri, M. J. Effect of nickel on combustion synthesized copper/fumed-SiO_2_ catalyst for selective reduction of CO_2_ to CO. *Int. J. Energy Res.* **46**, 441–451 (2022).

32. Zhu, Y. *et al.* Environment of Metal-O-Fe Bonds Enabling High Activity in CO_2_ Reduction on Single Metal Atoms and on Supported Nanoparticles. *J. Am. Chem. Soc.* **143**, 5540–5549 (2021).

33. Zhao, Z. *et al.* Atomically dispersed Pt/CeO_2_ catalyst with superior CO selectivity in reverse water gas shift reaction. *Appl. Catal. B Environ.* **291**, 120101 (2021).

34. Xin, H. *et al.* Overturning CO_2_ Hydrogenation Selectivity with High Activity via Reaction-Induced Strong Metal-Support Interactions. *J. Am. Chem. Soc.* **144**, 4874–4882 (2022).

35. Liu, H. *et al.* Ptn–Ov synergistic sites on MoO_x_/γ-Mo_2_N heterostructure for low-temperature reverse water–gas shift reaction. *Nat. Commun.* **13**, 5800 (2022).

36. Li, Y. F. *et al.* Cu Atoms on Nanowire Pd/H_y_WO_3-x_ Bronzes Enhance the Solar Reverse Water Gas Shift Reaction. *J. Am. Chem. Soc.* **141**, 14991−14996 (2019).

37. Li, Y. F. *et al.* Anchoring Ba^II^ to Pd/H_y_WO_3-x_ Nanowires Promotes a Photocatalytic Reverse Water–Gas Shift Reaction. *Chem. - A Eur. J.* **26**, 12355–12358 (2020).

38. Li, Y. F. *et al.* Pd@H_y_WO_3-x_ Nanowires Efficiently Catalyze the CO_2_ Heterogeneous Reduction Reaction with a Pronounced Light Effect. *ACS Appl. Mater. Interfaces* **11**, 5610–5615 (2019).

39. Wang, L. *et al.* Black indium oxide a photothermal CO hydrogenation catalyst. *Nat. Commun.* **11**, 2432 (2020).

40. Mine, S. *et al.* Reverse water-gas shift reaction over Pt/MoO_x_/TiO_2_: reverse Mars-van Krevelen mechanism via redox of supported MoO_x_. *Catal. Sci. Technol.* **11**, 4172–4180 (2021).

41. Wang, G. *et al.* Accelerated discovery of multi-elemental reverse water-gas shift catalysts using extrapolative machine learning approach. *ChemRxiv* 10.26434/chemrxiv-2022–695rj (2022).

42. Toyao, T., Kayamori, S., Maeno, Z., Siddiki, S. M. A. H. & Shimizu, K. Heterogeneous Pt and MoO_x_ Co-Loaded TiO_2_ Catalysts for Low-Temperature CO_2_ Hydrogenation to Form CH_3_OH. *ACS Catal.* **9**, 8187–8196 (2019).

43. Nie, L. *et al.* Enhanced performance of NaOH-modified Pt/TiO_2_ toward Room temperature selective oxidation of formaldehyde. *Environ. Sci. Technol.* **47**, 2777–2783 (2013).

44. Yu, J., Qi, L. & Jaroniec, M. Hydrogen production by photocatalytic water splitting over Pt/TiO_2_ nanosheets with exposed (001) facets. *J. Phys. Chem. C* **114**, 13118–13125 (2010).

45. Zhu, X., Cheng, B., Yu, J. & Ho, W. Halogen poisoning effect of Pt-TiO_2_ for formaldehyde catalytic oxidation performance at room temperature. *Appl. Surf. Sci.* **364**, 808–814 (2016).

46. Huang, H. & Leung, D. Y. C. Complete elimination of indoor formaldehyde over supported Pt catalysts with extremely low Pt content at ambient temperature. *J. Catal.* **280**, 60–67 (2011).

47. Zafeiratos, S., Papakonstantinou, G., Jacksic, M. M. & Neophytides, S. G. The effect of Mo oxides and TiO_2_ support on the chemisorption features of linearly adsorbed CO on Pt crystallites: An infrared and photoelectron spectroscopy study. *J. Catal.* **232**, 127–136 (2005).

48. Sener, C. *et al.* PtMo Bimetallic Catalysts Synthesized by Controlled Surface Reactions for Water Gas Shift. *ACS Catal.* **6**, 1334–1344 (2016).
